# Traditional utilization of bamboo in the Central Siwalik region, Nepal

**DOI:** 10.1371/journal.pone.0296886

**Published:** 2024-01-30

**Authors:** Bishnu Maya K. C., Janardan Lamichhane, Sanjay Nath Khanal, Dhurva Prasad Gauchan

**Affiliations:** 1 Department of Biotechnology, School of Science, Kathmandu University, Dhulikhel, Nepal; 2 Department of Environmental Science and Engineering, School of Science, Kathmandu University, Dhulikhel, Nepal; New York State Museum, UNITED STATES

## Abstract

Bamboo are the fastest growing perennial woody grasses that have versatile applications. Most of the local people inhabiting the riverine area of the Siwalik region of Nepal rely on bamboo products for economic benefits and medicinal uses. Our objective was to identify the diversity of bamboo species, their ethnomedicinal practices, and economic and ecological importance. Data were collected by direct observation, key informant interviews, participatory rural appraisal, inventory technique, focus group discussions, and a household survey using semi-structured and structured questionnaires. We recorded four genera and nine species of bamboo, of which eight species have been used for agriculture, five for medicine, four for construction, food, fodder, artifacts and religious purpose, three for river embankment, and two for ornamental purpose. As the local people in the study area were deprived of medical facilities, using traditional herbal medicine to cure various diseases was a common practice. The inhabitants responded that they use bamboo-based primary ethnomedicinal care even against snake and scorpion bites. Similarly, they use bamboo young culm for reducing body weight and control diabetes. The value of the informant consensus factor was found to be maximum for the bamboo against snake and scorpion bites (1.0) and minimum for weight loss (0.81). This study concludes that the traditional utilization of all kinds of bamboo in the region is vast despite their less diversity. The recorded bamboo species are used not only for food and fodder but also in preparing artifacts, soil nutrients restoration in the fallow land, construction materials for the rural people, river embankments, and religious and spiritual purposes. Therefore, if grown on a large scale, bamboo can provide sustainable benefits for the local users and ecological aspects. *Bambusa tulda* and *Dendrocalamus strictus* have a broad spectrum of pharmacological agents. Considering the multifaceted application of bamboo in the Siwalik area, it is worthwhile to encourage the local people to bamboo plantation, which would contribute to supplement their household requirements and be one of the alternative livelihood options.

## Introduction

Bamboo are ancient woody grasses and major non-timber forest products (NTFPs) that belong to the subfamily Bambusoideae of the grass family Poaceae [1]. They are found in the tropical, subtropical and temperate zones and are widely planted on private lands [2]. There are 121 genera and 1,662 species of bamboo in the world [3], of which Nepal harbours 23 genera and 81 species [4]. Bamboo popularly known as the “Green Gold of the forest” because of their varied applications [5]. They are equally important in the conservation of soil, water, and biodiversity and in the promotion of local and world economy as well [6]. The bamboo rhizosphere is a vital source of plant growth-promoting rhizobacteria (PGPR) [7] and a good source for sustainable bioenergy production through microbial fuel cells [8].

The history regarding the use of bamboo is intricately associated with humans from the time immemorial. According to Hinduism, Lord Krishna is associated with bamboo as he was fond of playinga flute made of bamboo [9]. Evidence shows that even Buddhist Monks carried bamboo seeds when they went to Japan from India with the aim to popularize Buddhism [10]. Owing to their expanding range of uses, bamboo have transformed from being known as "poor man’s trees" to "high-tech industrial raw materials" [11]. They have colossal use in construction, paper industries, scaffolding, agricultural farming, diesel, food, fodder, and preparation of musical instruments, medicines, aphrodisiacs, and varieties of household artifacts [12–14]. They have a higher raw material output than trees and high-profiting renewable resources [15]. Bamboo are characterized by low weight, tensile and compressive strength, elasticity and resistibility to physical damage, and can be harvested earlier (within 3–5 years) than trees which have potential usage only after 10–30 years [16]. According to Embaye et al. [17], industrial application of bamboo directly enhances the provision of income, food, survival and standard of livelihood for over 2.5 billion people worldwide. They have considerable potential as a wood substitute because they grow rapidly, and are over 20 times more sustainable in terms of their mechanical properties than timber, steel, and concrete [18]. Bamboo are also important entities of several natural and agricultural ecosystem services [19] and contribute to alleviating social and environmental problems in the developing world [18].

Developing bamboo resources as an eco-friendly raw material can help alleviate poverty, generate employment, and sustain rural livelihood [20]. Bamboo, an important component of the rural farming system of Tarai and Midhills of Nepal, serve as an alternate source of income as people are benefited from bamboo artifacts, fodder, fuel wood, construction materials, religious applications, and bamboo shoots used as vegetable and pickle [21]. Bamboo shoots have a long history of being used as a source of both food and medicine in China and Southeast Asia [22]. China is the richest country in bamboo resources, and Southeastern China alone accounts for 2/3^rd^ of the shoot yield and consumes more than 3/4^th^ of the total bamboo shoots produced [23]. The bamboo shoots are consumed in raw, canned, boiled, marinated, fermented, frozen, liquid, and medicinal forms [19] and can be processed into various beverages and medicines [24]. The medicinal property of bamboo, due to the presence of phytosterols and phenolic compounds, reportedly cures cardiovascular diseases, cancer, osteoarthritis, and osteoporosis [25]. The young shoots are rich in proteins, carbohydrates, minerals, fiber, and low in fat and sugars, improving appetite and digestion, and help in weight loss [26]. Thus, bamboo has also been drawing the attention of both health advocates and scientists worldwide.

Despite the established multiple benefits of bamboo, very few works have addressed the species diversity and economic importance of bamboo in the context of Nepal. Previous researches from Nepal have focused on the socioeconomic aspects of bamboo [27–31], its market potential [32], and extraction of phytohormone from bamboo seedlings [33]. To the extent of our literature search, the economic importance of bamboo was enumerated from only two partsof the country–Lalitpur and Rautahat [31, 32] and thus lacks significant utility regarding phytochemical and ethnobotanical importance of bamboo in Nepal. Therefore, this study aimed to identify the species diversity of bamboo, their traditional utilization, ethnomedicinal practices, and the economic importance in the central Siwalik region of Nepal. This study has also discussed the future prospects of bamboo to strengthen the alternative livelihood options for the local users.

## Materials and methods

### Ethical approval

The study was conducted in the Central Siwalik Region of Nepal. The President Chure Tarai Madhesh Conservation and Development Board (PCTMCDB), Khumaltar, Lalitpur, Nepal,had already taken consent from the district authority to organize a field-based study in the seven districts of the study area. Besides this, informed consent was taken from the local community authority to grant permission for field visits and a round of questionnaire survey in every sampling site of each district selected for the study. During the field visit, the importance of the study was described to all the stakeholders by organizing a community-based meeting. Additionally, for the questionnaire survey, verbal consent was obtained from the selected respondents and the field assistants at the witness of the community authority and other responsible persons. None of the children were involved in this study. The respondents were also informed that they could withdraw their consent at any time during the study period.

### Study area

**Fig 1:**
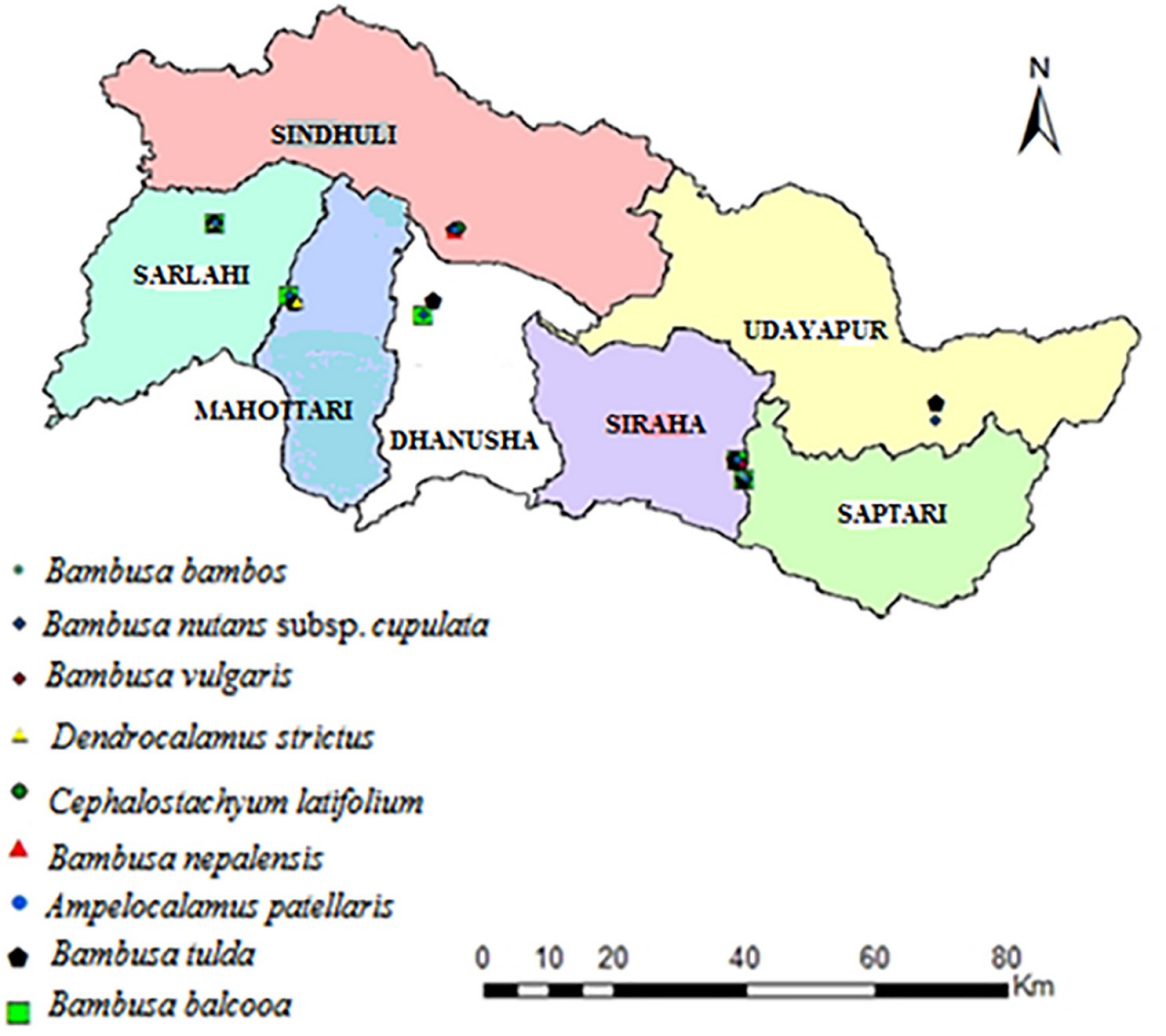
Location of sampling sites.

This study was carried out in seven districts (Udaypur, Saptari, Siraha, Dhanusha, Mahottari, Sarlahi and Sindhuli) situated within the central Siwalik region of Nepal (Fig 1). Siwalik region, also popularly known as Chure or Churia, the youngest hills formed by depositing river products around 40 million years ago, extends from the Indus river of Pakistan in the West to Brahmaputra river of India in the East. About 12.78% of the total land of Nepal lies in the Siwalik region [34]. The Siwalik land is geographically very rugged, soft, and unstable, consisting of tertiary unconsolidated and highly erodible fluvial sediments [35]. In this region, the temperature varies from 28.4°C to 35.9°C in June and from 5°C to 11.10°C in January [36]. Various ethnic communities like *Dome*, *Mushahar*, *Majhi*, *Kumal*, *Tamang*, *Magar*, *Chhetri*, and *Brahman* are dominant in this region.

### Ethno-ecological and household survey technique

This research is the outcome of two-years field survey (2016–2018). The site selection was based on the priority made by PCTMCDB for the sustainable conservation of the Siwalik region by bamboo plantation. The PCTMCDB Khumaltar, Lalitpur, Nepal, and Kathmandu University (KU) Dhulikhel, Kavre, Nepal signed the memorandum of understanding (MoU) explicitly mentioning the research objectives. Altogether, 210 informants (155 male and 55 female) representing different age groups from 18 ethnic communities of seven districts (Tables 1 and 2) were includedin the study. The information regarding the ethnobotanical utilization of bamboo were gathered through semi-structured and structured questionnaires. A household survey was conducted among the local residents, ethnic communities, primary traders, secondary traders, local artisan groups, local vendors and traditional medicinal practitioners.

**Table 1:** Major characters of the study area.

Districts	Sampling sites	Nearest trade Centre	Physiography	Major ethnic groups
**Udaypur**	Hadiya	Gaighat	Siwalik	*Tharu*, *Brahman*, *Magar* and *Chhetri*
**Saptari**	Tikulia	Lahan, Rajbiraj	Siwalik	*Yadv*, *Mandal* and *Tharu*
**Siraha**	Naudega	Lahan, Siraha	Siwalik	*Tharu*, *Mandal*, *Thakur*, *Mushahar*, *Paswan*, *Chamar* and *Sudi*
**Dhanusa**	Aauribaba	Dhalke, Janakpur	Siwalik	*Yadav*, *Mahato*, immigrated *Magar*
**Mahottari**	Laxminiya	Kantibazar, Gausala, Jaleswhor	Siwalik	*Yadav*,*Mahato*, *Mushahar*, *Dome*, *Mandal* and *Magar*
**Sarlahi**	Sasapur	Nawalpur, Lalbandhi	Siwalik	*Tamang*, *Bhujel*, *Dome*, *Chhetri*, *Magar*, *Pariyar*
**Sindhuli**	Mathillo Ranibans	Sindhuli bazar, Bardibans	Siwalik	*Majhi*, *Magar*, *Kumal*, *Hyawu* (Endangered ethnic group), *Brahman* and *Chhetri*

**Table 2:** Demographic characteristics of the respondents involved in the study during the field visit.

S. No.	Ethnic Groups	Districts							Total respondents (%)	Gender		Age groups (Years)					Occupation				Education				
		Udayapur	Saptari	Siraha	Dhanusha	Mahottarai	Sarlahi	Sindhuli		M	F	20–30	31–40	41–50	51–60	˃ 60	Government job	Farmer	Artisan	Fishing	Illiterate	Primary	Lower secondary	Secondary	University level
1	*Bhujel*	-	-	-	-	-	9	-	9	3	6	2	2	-	4	1	-	9	-	-	1	7	1	-	-
2	*Bramhan*	2	-	-	-	-	-	7	9	6	3	2	-	5	2	-	2	7	-	-	2	3	-	4	-
3	*Chamar*	-	-	-	-	1	2	-	3	3	-	-	1	-	2	-	-	-	3	-	3	-	-	-	-
4	*Chhetri*	4	-	-	-	2	-	2	8	6	2	-	3	3	-	2	-	8	-	-	2	4	2	-	-
5	*Dome*	-	5	-	-	10	12	-	27	19	8	3	8	-	12	4	-	-	27	-	27	-	-	-	-
6	Hyawu*	-	-	-	-	-	-	2	2	2	-	-	1	-	1	-	-	2	-	-	1	-	1	-	-
7	*Kumal*	6	-	-	-	-	-	12	18	14	4	2	12	3	1	-	1	12	-	5	8	4	5	1	-
8	*Magar*	-	-	-	4	2	-	-	6	4	2	1	2	-	3	-	1	5	-	-	4	1	-	-	1
9	*Mahato*	-	-	-	4	6	-	-	10	9	1	3	5	1	-	1	3	7	-	-	2	4	1	1	2
10	*Majhi*	2	-	-	-	-	-	10	12	4	8	4	3	2	1	2	2	3	-	7	7	3	1	1	-
11	*Mandal*	-	7	4	-	1	-	-	12	10	2	-	6	2	4	-	-	8	4	-	12	-	-	-	-
12	*Mushahar*	-	-	3	-	12	-	-	15	14	1	4	3	5	2	1	-	-	15	-	15	-	-	-	-
13	*Pariyar*	3	-	-	-	1	2	4	10	7	3	2	3	3	1	1	-	7	3	-	8	2	-	-	-
14	*Paswan*	-	4	5	2	-	-	-	11	7	4	3	6	1	1	-	-	11	-	-	10	1	-	-	-
15	*Tamang*				2	2	14		18	13	5	-	8	4	2	4	-	18	-	-	8	6	4	-	-
16	*Thakur*	-	3	2	3	4	-	-	12	11	1	2	5	3	2	-	5	7	-	-	2	1	3	4	2
17	*Tharu*	5	-	3	-	-	4	-	12	9	3	2	5	2	2	1	-	12	-	-	8	2	2	-	-
18	*Yadav*	-	6	5	2	3	-		16	14	2	4	5	3	3	1	6	10	-	-	5	3	2	3	3
Total	210	22	25	22	17	34	25	37	210	155	55	34	78	37	43	18	20	126	52	12	125	41	22	14	8

*Endangered ethnic group

### Focus group discussion (FGD)

**Fig 2:**
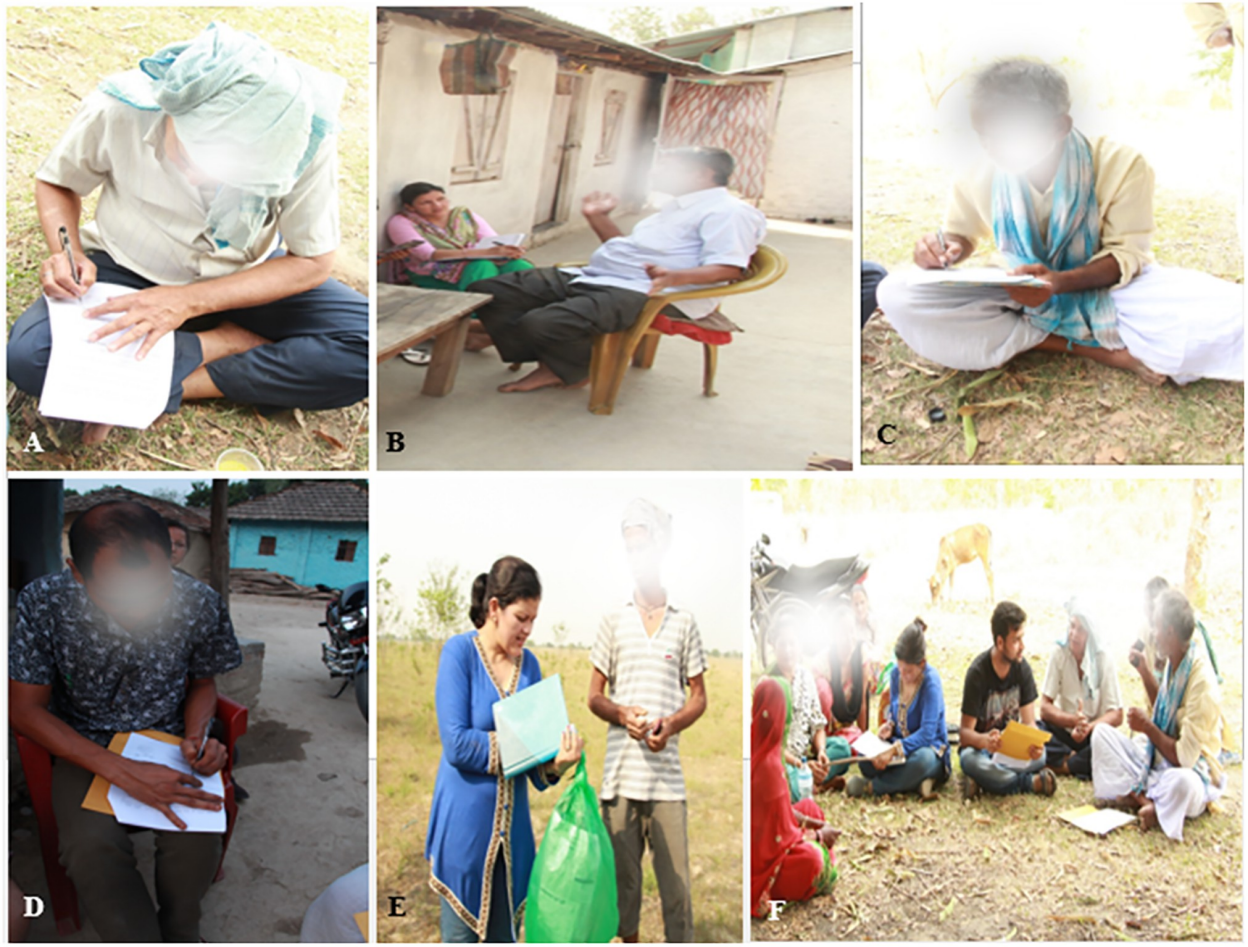
Questionnaire survey: A, B, C and D- Key informants, E- Interview with forester, and F- Focus group discussion.

Focus group discussion (FGD) was conducted by following the participatory rural appraisal (PRA) method [37]. FGD was organized among various local groups such as community forest user groups, women groups, forest officers, healers, traders, indigenous communities, bamboo artisan groups and immigrated communities (Fig 2F). For this purpose, structured and semi-structured questionnaires (S1 Table) were prepared following Edwards et al. [38] and forwarded to the focus groups. Altogether 35 focus group discussions were carried out (five from each district). Each group was asked about the local name of bamboo plants, parts used, forms of use, purpose of use, location, place of availability, growing condition, commercial status, harvesting methods, conservation aspect and socioeconomic status.

### Key informant interview (KII)

Key informant interview KII was conducted according to Kumar [39]. All the informants were informed regarding the background of the research objectives prior to the interview. To avoid scheduling conflict, an earlier appointment was arranged with the key informants. Altogether 63 key informants (nine from each district) who were able to communicate their ideas, views and opinions about the utilization of bamboo and their livelihood support were included (Fig 2A–2E) for KII. The key information was obtained from various groups of people like local bamboo artisan group, farmers, healers, dhami (practitioners engaged in alleviating illness based on spiritual believes), bamboo traders, women, forest officers, and head of the immigrated and indigenous communities.

### Inventory technique

Inventory technique (IT) [37, 40] comprised of a collection of different bamboo specimens from the nearby sampling sites and enlisting the associated ethnobotanical knowledge. These information were collected through the identification of their local name, parts used, the purpose of use, location, growing condition, availability and conservation status since ten years (Fig 2E). At the same time, in-depth market assessments were conducted at local markets to identify the available bamboo artifacts, their market value, status and trends in use from the standpointof the purchasers and sellers.

### Ranking of socioeconomic status of bamboo user groups (BUGs)

The privileged, underprivileged and deprived BUGs were identified with the help of PRA tool based on income status and land ownership criteria. All household names were written, and the participants of BUGs were asked to rank them according to the overall landholding conditions, income source, amount of saving and/or loan. The information was cross checked with the same users while they were invited in gathering with the villagers. The categories used to classify the BUGs particularly in this study have been provided in Table 3.

**Table 3:** Ranking of socioeconomic status of BUGs.

Privileged class	Underprivileged class	Deprived class
They are able to feed their family members throughout the year from their own agricultural production.	They are able to feed their family members for four to six month in a year from their own agricultural production.	They are unable to feed their family members for less than one month in a year from their own agricultural land.
They have enough land (Six kattha to three biga)	They have little land (Five dhur to one kattha)	They have less than five dhur land
They provide land on rent.	Work as labor in other land owners house or farms	Fully depend on bamboo artifacts.
Example: *Brahman*, *Chettri*, *Yadav*, *Mahatto*, *Chaudhary*	Example: *Magar*,*Kumal*, *Tamang*, *Mandal*	Example: *Dome*, *Mushahar*, *Chamar*

Note: 1Bigha = 6772.63 m^2^;1Kattha = 338.63 m^2^;1; Dhur = 16.93 m^2^

### Plant collection, identification and preservation

**Fig 3:**
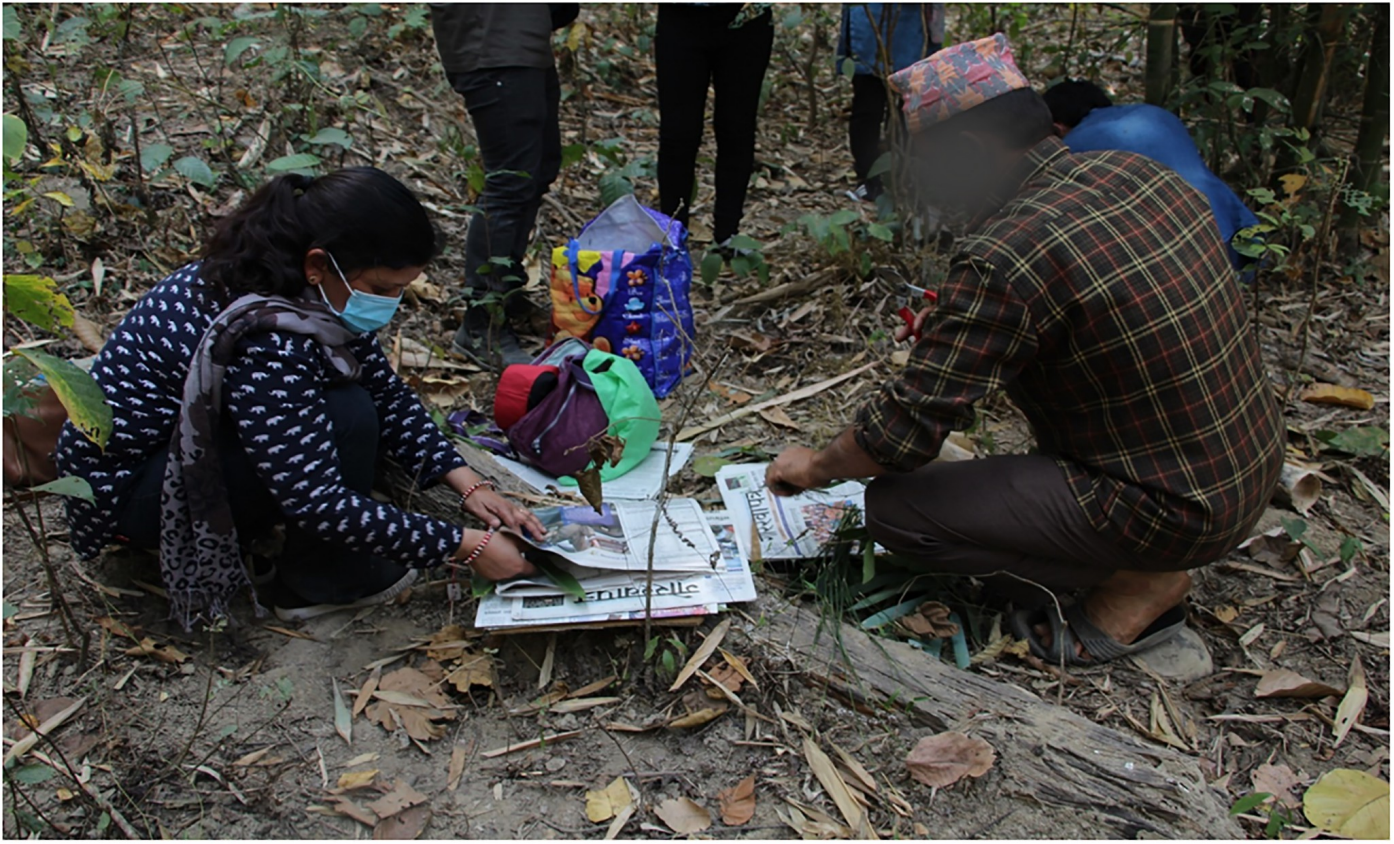
Researcher (Left) preparing herbarium of collected bamboo specimens.

The voucher specimens of bamboo for herbarium preparation were collected following the guidelines provided by PCTMCDB. Organic Farming and Natural Product Research Centre (ONRC) of School of Science, Kathmandu University led the entire fieldwork. The specimens were collected in duplicate and information regarding ethnobotanical utilization of bamboo was assembled during the field visit (Fig 3). The collected bamboo specimens were identified using the published literature [4, 29, 41–45]. The herbarium specimens were prepared according to Jain and Rao [46] and tagged with information like altitude, latitude, longitude and locality of collection. The voucher specimens of identified bamboo were deposited at ONRC.

### Post-field visit authentication of bio-efficacy of selected bamboo species

After the first and second field visits were completed, the collected information was discussed with the research team, district and local level stakeholders, and bamboo experts. A brief post-field survey was carried out to gather some missing information, confirm local people’s ethnomedicinal practices, and clarify and comprehend some discrepancies in the information collected during the previous visits. The ethnomedicinal properties of selected bamboo species were compared with information obtained from the ethnic communities and the review of the available literatures for all species for which information was available.

### Statistical analysis

Data regarding the availability, parts used, purpose of utilization, voucher specimen number, local name and altitude were recorded and analyzed using descriptive analysis, and results were presented systematically in tabular form. Informants consensus factor (ICF) was calculated according to Heinrich et al. [47] to find out the homogeneity in the information provided by the informants.

$$Informants\;consensus\;factor\;(ICF)=\frac{nur-nt}{nur-1}$$

Where, ‘nur’–total number of use report for each disease cluster; ‘nt’–total number of species used for that cluster.

## Results

### Livelihood status of ethnic community of BUGs in the study sites

Agriculture is the major source of livelihood for the majority of households (92%) who mainly produce rice, wheat, maize, sugarcane and potato as the major crops. However, only 2% of the households were able to meet all their annual food requirements from their own production but for the rest of the inhabitants, the food was said to be sufficient for two months or less. To meet the food requirements during the months of food deficit, the people responded to adopt multiple coping strategies such as seasonal migration for work to the district headquarters and various parts of India, and selling collected bamboo young shoots (commonly known as *tama* in Nepali language) as well as bamboo artifacts. *Dome*, *Majhi*, *Mushahar* and *Kumal* people are landless and rely on bamboo and bamboo products, while in rainy season, the *Majhi* community depend on fishing for their daily requirements. Similarly, *Tamang*, *Bhujel*, *Magar*, *Yadav*, *Mahato*, *Chaudhary*, *Chettri* and *Brahman*, who own at least some land for agriculture, partially depend on bamboo and bamboo products. Of the total informants, 90.47% (n = 190) knew at least a single use of bamboo.

### Distribution of bamboo species and pattern of ethnobotanical utilization

**Fig 4:**
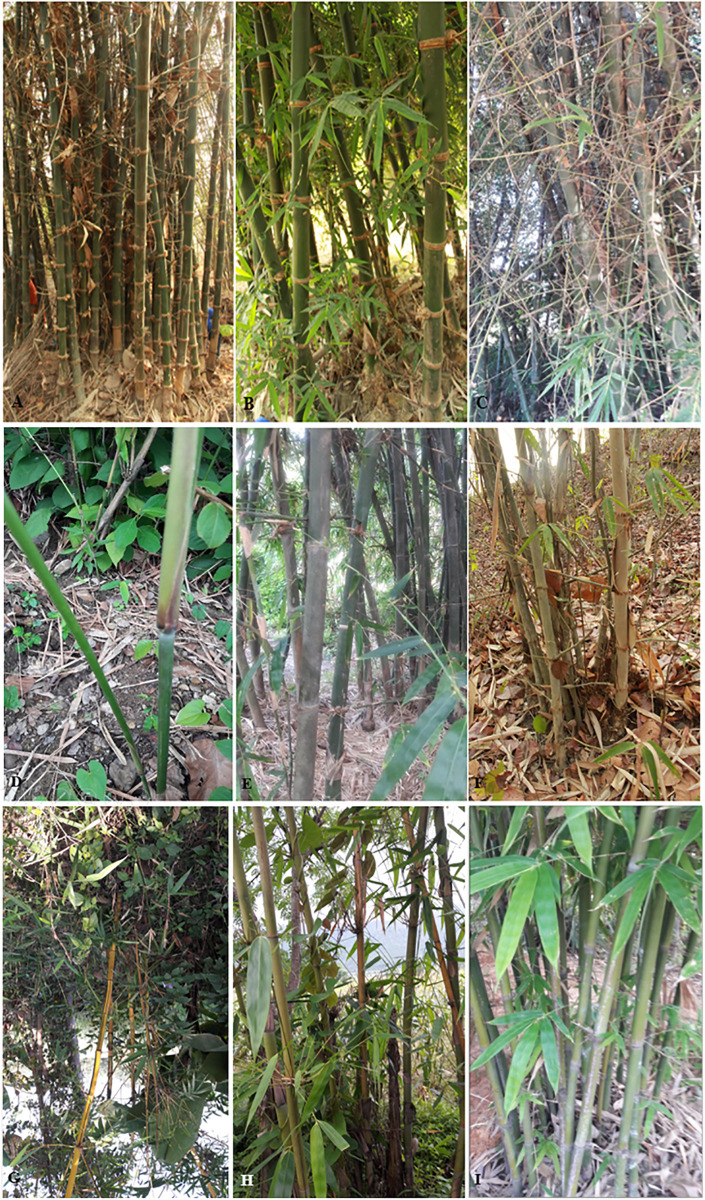
Bamboo species distributed in the study area: A- *Bambusa tulda*, B- *B*. *balcooa*, C- *B*. *bambos*, D- *Ampelocalamus patellaris*, E- *B*. *nutans* subsp. *cupulata*, F- *B*. *nepalensis*, G- *B*. *vulgaris*, H- *Cephalostachym latifolium* and I- *Dendrocalamus strictus*.

Among 21 genera and 81 species of bamboo distributed in various parts of the country, altogether 4 genera and 9 species of bamboo have been reported and collected from our study areas (Fig 4A–4I).

**Fig 5:**
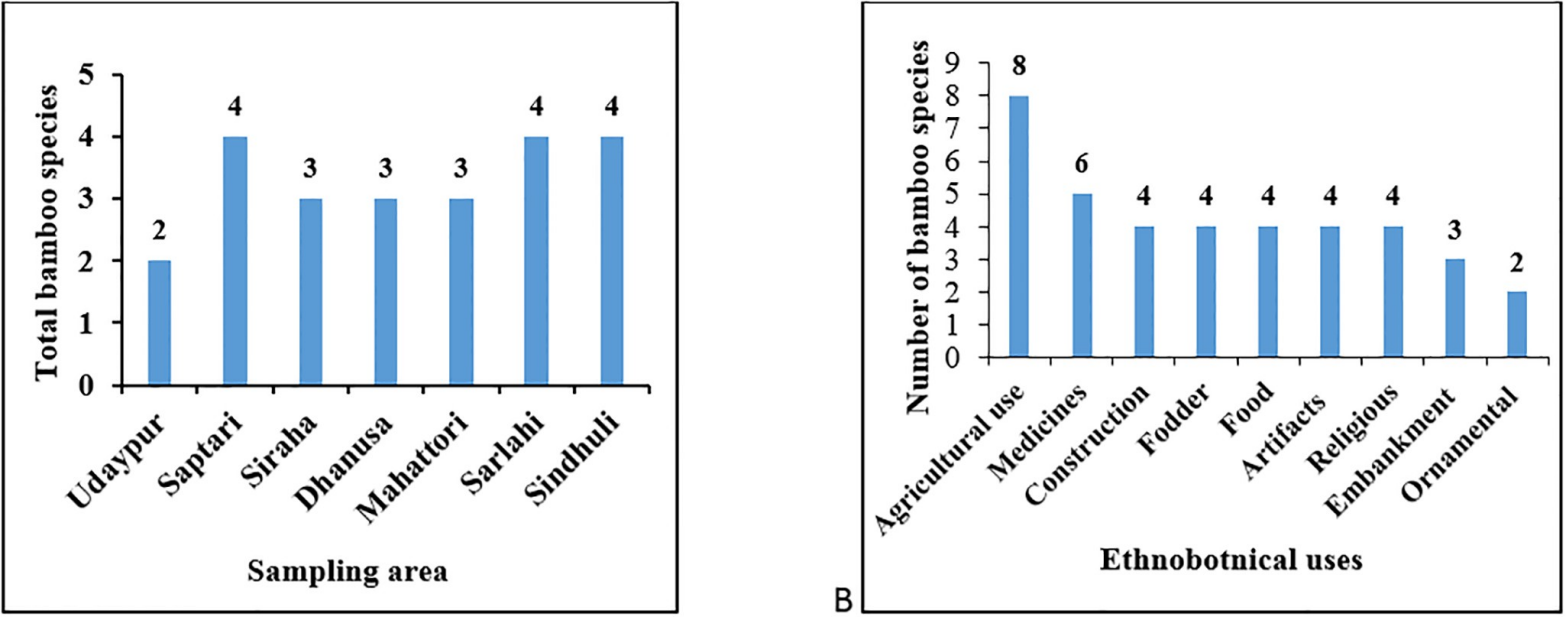
A-Total number of bamboo species in sampling sites. B-Major uses of bamboo species recorded from the study sites.

Only two bamboo species were recorded from Hadiya of Udaypur, three species each from Laxminiya of Mahottari, Naudega of Siraha and Aauribaba of Dhanusa and four each from Tikulia of Saptari, Sasapur of Sarlahi and Mathillo Ranibans of Sindhuli (Fig 5A).

Based on the survey of local communities, it was witnessed that a variety of products were made from bamboo. This was their indigenous knowledge acquired from their seniors (Table 4). However, the number of bamboo products made per household varies considerably. There were more bamboo fencing (*bei*) in each household than other bamboo products.

**Table 4:** Bamboo distribution and uses in Central Siwalik region, Nepal.

Bamboo species	Vernacular name	Voucher specimen	Distribution	Ethnobotanical uses
*Ampelocalamus pattelaris* (Gamble) Stapleton	Nibha bans, Lyabans	25-04-2017 BMKC, 1577 (KU)	**Sindhuli**: (N 27°04’23.6’’, E 086°01’11.9’’, 329m).	Fodder, construction, food.
*Bambusa balcooa* Roxb.	Haroth	19-05-2016, BMKC, 1561 (KU)	**Sarlahi**: (N 27°05’02.5’’ E 085°37’002’’, 143m);	Food, construction, fodder, basket, wall floor, agricultural use (support to creepers and/or climbers, bar to protect the entry of unwanted animals), fencing, bamboo pole, game (*dhanush*), anticoagulant.
			**Mahottari**: (N 26°57’48.2’’ E 085°44’32.1’’, 128m);	
			**Dhanusha**: (N 26°55’45.6’’, E 085°58’06.2’’, 166m);	
			**Siraha**: (N 26°41’10.8’’, E 086°29’45.3’’, 94m);	
			**Saptari**: (N 26°39’13.8’’, E 086°30’35.6’’, 80m);	
			**Nepal**: (WCE, 80-1600m).	
*Bambusa bambos* Wild.*	Kande bans	17-02-2018, BMKC, 1599 (KU)	**Saptari**: (N 26°39’15’’, E 086°30’17.2’’, 80m);	Construction, fencing, hedge, agricultural use (support to creepers), medicine for snake bite.
			**Nepal** (EC,50-1463m)	
*Bambusa nepalensis* Stapleton	Tama bans, Phusre bans	25-04-2017, BMKC, 1575 (KU)	**Sindhuli**: (N 27°04’24.1’’, E 086°01’11.7’’, 315m);	Fodder, food, woven products, ropes, vegetable, pickle.
			**Nepal**: (ECWMW, 300-1500m).	
*Bambusa nutans* subsp. *cupulata* Stapleton	Mal bans	25-06-2017, BMKC, 1579 (KU)	**Sarlahi**: (N 27°05’03.9’’, E 085°36’59.9’’, 138m);	Construction, food, scaffolding, woven products (mat, basket, *doko*/*thunche*, *dhakiya*, *terango*, *gidra*, *mahalo*/*makhara*, *ghum*, *bhakari*, tray, dust bin, *ghiri*), *dhungro*, fodder, tooth brush, medicines (abdominal disorder in livestock and nocturnal enuresis for children), decoration (cultural / religious ceremonies).
			**Mahottari**: (N 26°57’49.4’’, E 085°44’29.2’’, 121m);	
			**Dhanusha**: (N 26°55’46.5’’, E 085°58’5.6’’, 170m),	
			**Siraha**: (N 26°41’11.06’’, E 086°29’42.9’’, 94m);	
			**Saptari**: (N 26°39’16.4’’, E 086°30’36.4’’, 72m);	
			**Sindhuli**: (N 27°04’24.3’’, E 086°01’11.7’’, 316m);	
			**Udayapur**: (N 26°46’55.4’’, E 086°49’51.7’’, 113m);	
			**Nepal**: (ECMW, 70-1500m).	
*Bambusa tulda* Roxb.	Champ bans, Kanda bans	20-04-2017 BMKC, 1563 (KU)	**Sarlahi**: (N 27°05’02.6’’, E 085°36’59.8’’, 140m);	Vegetable, pickle and local alcohol from fermented young culm, agricultural use (support to creepers), fencing, housing, ropes, trays, *bei* (for plant protection), *chalno*, pen box, dust bin, tray, lampshed, hanger, window.
			**Mahottari**: (N 26°57’07.5’’, E 085°45’03.9’’, 115m);	
			**Dhanusha**: (N 26°57’17.5’’, E 085°59’04.2’’, 204m);	
			**Udayapur**: (N 26°46’54.9’’, E 086°49’55.6’’, 111m);	
			**Siraha**: (N 26°41’00.6’’, E 086°29’39.1’’, 93m);	
			**Saptari**: (N 26°39’16.4’’, E 086°30’35.9’’, 74m);	
			**Nepal**: (CMW, 70-204m).	
*Bambusa vulgaris* Vittata*	Pahelo bans	11-05-2018 BMKC, 1591(KU)	**Siraha**: (N 26°40’43’’, E 086°30’18’’, 94m);	Fodder, ornamental use, toothpick, medicine for snake bite.
			**Nepal**: (EC-80, 1200m).	
*Cephalostachyum latifolium* Munro.	Gopi bans	26-06-2016 BMKC, 1590 (KU)	**Sindhuli**: (N 27°04’42.3’’, E 086°01’38.4’’, 325m);	Fencing, ornamental use, garden ropes.
			**Nepal**: (ECMW, 300-2000m).	
*Dendrocalamus strictus* (Roxb.) Nees	Latthi bans, Kath bans	15-10-2017 BMKC, 1598 (KU)	**Sarlahi**: (N 27°05’01.6’’, E 085°36’41.8’’, 140m);	Driving animals, fodder.
			**Mahottari**: (N 26°57’08.5’’, E 085°45’06.6’’, 115m);	
			**Nepal**: (EC, 80-205m).	

*Species not recorded in Annotated Cheklist of the Flowering Plants of Nepal. WCE: Western, Central and Eastern Nepal, EC: Eastern and Central Nepal, ECMW: Eastern, Central and Mid-western Nepal, CMW: Central and Mid-western Nepal, BMKC: Bishnu Maya KC (Specimen collector); KU: Kathmandu

Our study revealed that all types of bamboo were found being utilized by the local community to meet their daily requirements. The pattern of use of bamboo such as agricultural use (AU), artifacts use (ARTU), construction use (CU), fodder use (FDU), food use (FU), medicinal use (MU), ornamental use (OU), religious use (RU) and embankment use (EMBU) vary between privileged, underprivileged and deprived communities in the study sites (Fig 5B). Deprived communities lack bamboo raw materials from their own production as they do not possess their land for bamboo plantation, and therefore have to invest most of their time for searching bamboo raw materials, harvesting and transportation from distant villages.

Questionnaire survey to the informants under three categories (Table 5) revealed that the bamboo utilisation pattern was fewer in deprived communities. The houses of all the deprived people were found to be constructed exclusively from bamboo, 98.90% of them had used bamboo for making artifacts and religious purpose, 69.66% answered that they use bamboo for medicinal purposes and 52.74% used young bamboo shoot for their food. The majority of the privileged (92.85%) and under privileged (96.10%) people used bamboo for varieties of agricultural purposes. It was a common observation in all kinds of communities that more than 95% of them use bamboo for various religious purposes. As the privileged and under privileged people have their own land, they use bamboo for several agricultural purposes, construction of embankment to mitigate the damage of their agricultural field from flood and landslide, and fodder to feed their cattle. Moreover, the plantation of ornamental bamboo in the courtyard and use of bamboo artifacts for decoration in the houses of privileged and under privileged people was found to be a common trend.

**Table 5:** Pattern of utilization of bamboo by BUGs.

Uses of bamboo	Percentage of BUGs sharing benefits		
	Privileged	Under privileged	Deprived
AU	92.85	96.10	-
ARTU	23.80	27.27	98.90
CU	9.52	46.75	100
EMBU	52.38	92.20	-
FDU	80.95	80.51	-
FU	45.3	66.23	52.74
MU	9.52	27.27	69.66
OU	76.19	72.72	-
RU	95.23	97.40	98.90

BUGs: Bamboo user groups, AU-Agricultural uses, ARTU: Artifacts use, CU: Construction use, EMBU: Embankment use, FDU: Fodder use, FU: Food use, MU: Medicinal use, OU: Ornamental use; RU: Religious use.

**Fig 6:**
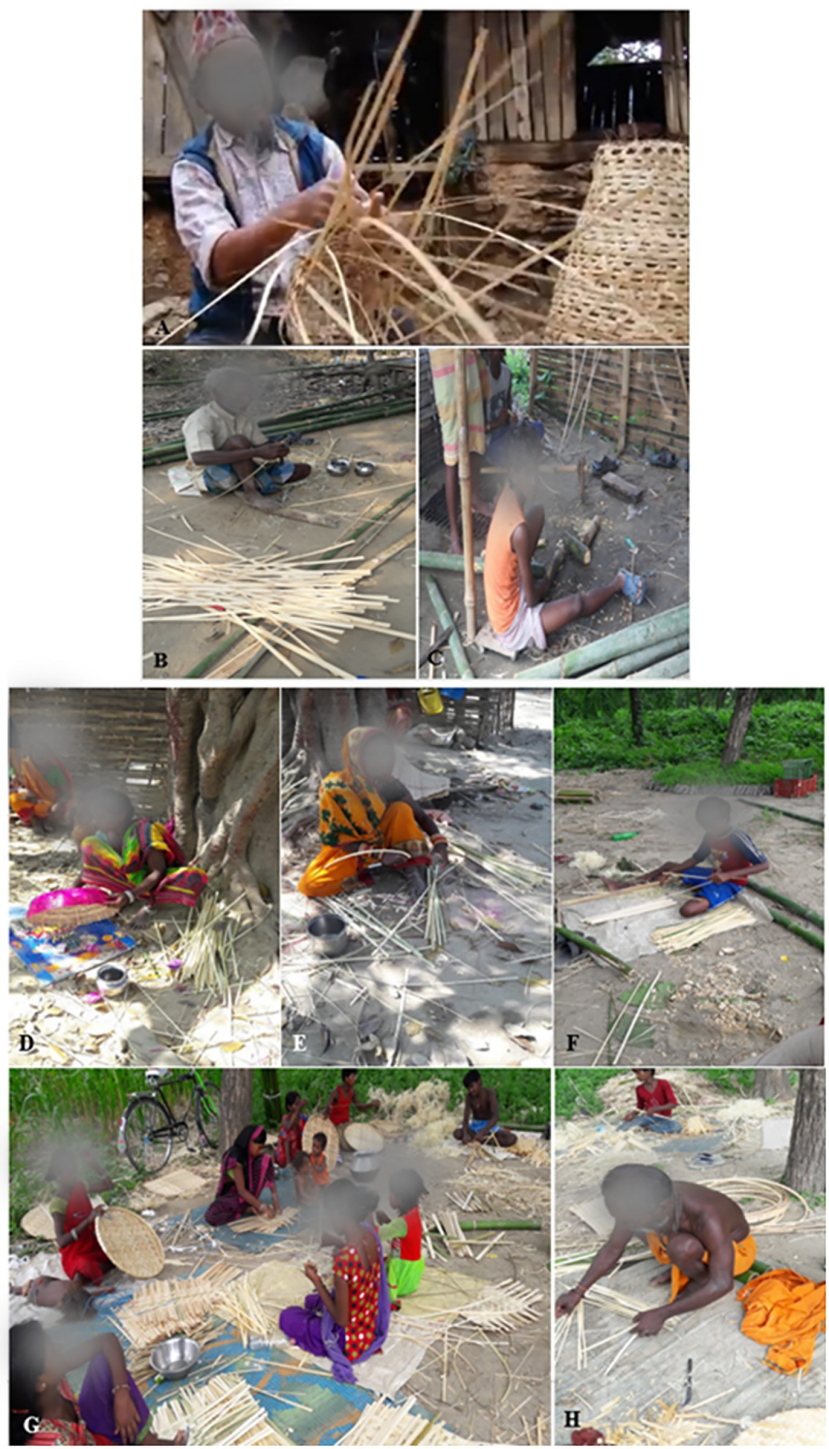
Bamboo artisan group preparing various bamboo artifacts (A- Preparing *doko*, B- Preparing bamboo splits (*choya*), C- Cutting bamboo culms for making splits (*choya*), D- Painting *dhakiya*, E- Arranging bamboo splits (*choya*), F- Cutting bamboo splits (*choya*) for preparation of hand-fan (*hate pankha*), G- Preparing bamboo mat (*mandro*) and *supli*, and H- Arranging bamboo splits (*choya*) for preparing bamboo mat.

Respondents in the study area use bamboo species to meet their daily basic requirements. According to local bamboo artisan groups (Fig 6A–6H), they select certain types of bamboo depending on what kind of artifacts they need to prepare. The majority of bamboo species (eight species) in our study sites were utilised for agricultural purposes except *A*. *patellaris*, which is mainly used for fodder for goat and cattle because this species has thin culm with small branches and large leaves.

Regarding the uses of bamboo-based on their age, the bamboo less than one year age (young bamboo shoot i.e. *tama*) is mostly used for vegetables, pickles and preparation of local alcoholic beverages by fermentation. Varieties of artifacts are prepared by the bamboo artisan groups from the bamboo splits (*choya*) produced from the bamboo of the age group 1–3 years, and various weaving materials, bamboo sticks (for driving animals and walking), *dhungro*, *ghiri*, dustbins are prepared from bamboo of four to seven years of age. Most of the agricultural uses of bamboo for making fencing around the crop field are obtained from mature bamboo of the age group four to seven years. The bamboo of more than seven years are considered very strong and they are used for the construction of houses, furniture, fencing and embankment. Similarly, the dead culms are used as firewood and charcoal (Table 6).

**Table 6:** Age wise utilization of bamboo culm.

Bamboo species	Bamboo age classes and their uses				
	<1 year	1-3year	4–7 year	>7year	Dead culms
*A*. *pattelaris*	-	Fodder, agricultural purpose	Agricultural purpose	Fencing, agricultural purpose	Firewood
*B*. *balcooa*	Vegetable, pickle, local alcohol	Weaving materials (basket, *doko*, tray and rope),medicines	Weaving materials for granaries	Building poles for houses and fencing, embankment	Firewood and charcoal
*B*. *bambos*	-	Agricultural purpose, medicine	Agricultural purpose	Building poles for houses and fencing, embankment	Firewood and charcoal
*B*. *nepalensis*	Vegetable, pickle, medicine	Basket, *torungo*, *doko*, tray, dust bean, rope	Weaving materials for granaries and beehives	Building poles for houses and fencing	Firewood and charcoal
*B*. *nutans* subsp. *cupulata*	Vegetable, pickle, local alcohol,medicines	Baskets, *torungo*, *doko*, tray, dust bean, rope, *jyaphri* (window), Nepali umbrella (*ghum*), *mahala*	Weaving materials for granaries and beehives	Building poles for houses and fencing, lamp shed	Firewood and charcoal
*B*. *tulda*	Vegetable	*Thunche*, *bhakari*, dust bean	Weaving materials for granaries, *ghiri*, *dhungro*	Building poles for houses and fencing, agricultural purpose, embankment	Firewood and charcoal
*B*. *vulgaris*	None	Ornamental use, medicines	Ornamental use	Fencing, agricultural purpose	Firewood
*C*. *latifolium*	Vegetable, pickle, local alcohol, medicines	Tooth picks, rope, dust bean, vase	Dust bean, tray, vase, stick, *gidra*, *torungo*	fencing, photo frame, Embankment	Firewood
*D*. *strictus*	Medicines	Fodder, medicines	Animals driving stick, walking stick	Furniture (sofa, bed, table, chair) and fencing	Firewood

### Bamboo used as medicines

In response to the question regarding the prevalent diseases in study areas, key informants responded that dysentery, diarrhoea, jaundice, typhoid, gastritis, hypertension, and diabetes are common diseases in their communities. Besides these, snake and scorpion bites arecommon experiences of the local people, especially during the summer. According to the responses to the questionnaires about traditional medicinal practices, they were found using certain species of bamboo (*B*. *bambos*, *B*. *tulda*, *B*. *nutans* subsp. *cupulata*, *D*. *strictus*, *B*. *nepalensis* and *B*. *balcooa*) in addition to other common medicinal plants. About 70.0% of the respondents from deprived communities use bamboo for medicines, 27.27% of the respondents from underprivileged and 9.52% from privileged communities mentioned the uses of bamboo as an antiseptic and healing agent for external injuries in the human body. Hairs of culm sheath of *D*. *strictus*, *B*. *nutans* subsp. *cupulata*, *B*. *balcooa* and *B*. *tulda* are used to make powder to be applied externally on freshcuts and wounds (Table 7). A local healer from Sarlahi (Mr. Jogbahadur Moktan, age 54 in personal conversation) replied that he got this knowledge from his father, and he believed this practice has been proved by applying powder of hairs of bamboo culm sheath around the injured portion. However, this information was not found in other study sites. Collected bamboo sap in the inter-nodal region (commonly known as *bansko dhungra ko paani*) of *B*. *nutans* subsp. *cupulata* and *B*. *tulda* is used to cure the nocturnal enuresis (bed wetting) as well as jaundice. The decoction of a young shoot of *B*. *bambos* is used to cure sores and that of *D*. *strictus*, *B*. *nepalensis* is known to be a good tonic for cough and respiratory disorders.

**Table 7:** Comparison between phytochemical properties and indigenous uses of bamboo.

Bamboo species	Phytochemical properties (Literature review)	Indigenous uses (Present study)	Method of use
*B*.*balcooa*	Contains several phytochemicals including tannins, saponins, steroids, flavonoids, phenolic compounds [48–50]	• Maintaining blood sugar and reducing body weight	• Boiled young culm
*B*.*tulda*	Contains phenolic compounds,flavonoids, and antimicrobial properties [51]	• Nocturnal enuresis	• Two spoonful internal sap is taken twice a day for curing
		• Jaundice	• One spoonful sapmixed with one spoonful decoction of *Cuscuta reflexa* is taken once a day
*B*.*nutans* subsp.*cupulata*	Antioxidant and antimcrobial properties [52, 53]	• Nocturnal enuresis	• Two spoonful internal sap taken twice a day
		• Jaundice.	• One spoonful sap mixed with one spoonful decoction of *Cuscuta reflexa*is taken once aday
		• Oral infection.	• Sub branches are used for making brush inorder to prevent
*B*.*nepalensis*	Antimicrobial properties,Phenolic compounds [54]	• Skin rejuvenation	• About 200gm of the fresh young culm is boiled with water containing few amount of turmeric and salt and taken once a week
*B*. *bambos*	Contains several antioxidant compounds like betaine [55]	• Snake and Scorpion bite.	• Leaf paste is applied at the injured part
		• blood sugar and reducing weight	• Boiled young culm
		• Sore throat	• One spoonful decoction of young culm with salt is taken twice a day for two to three days
*D*.*strictus*	Contains antioxidants compounds like flavonoids and phenolic compounds, antimcrobial properties [56]	• Respiratory disorder	• Decoction of young culm
		• Cut and wounds	• About 250gm flesh of young culm is boiled in water and mixed with decoction of *Acorus calamus* and two spoonful is taken thrice a day for 4 to 6 days
			• Culm hair powder applied on fresh cuts or wounds

**Fig 7:**
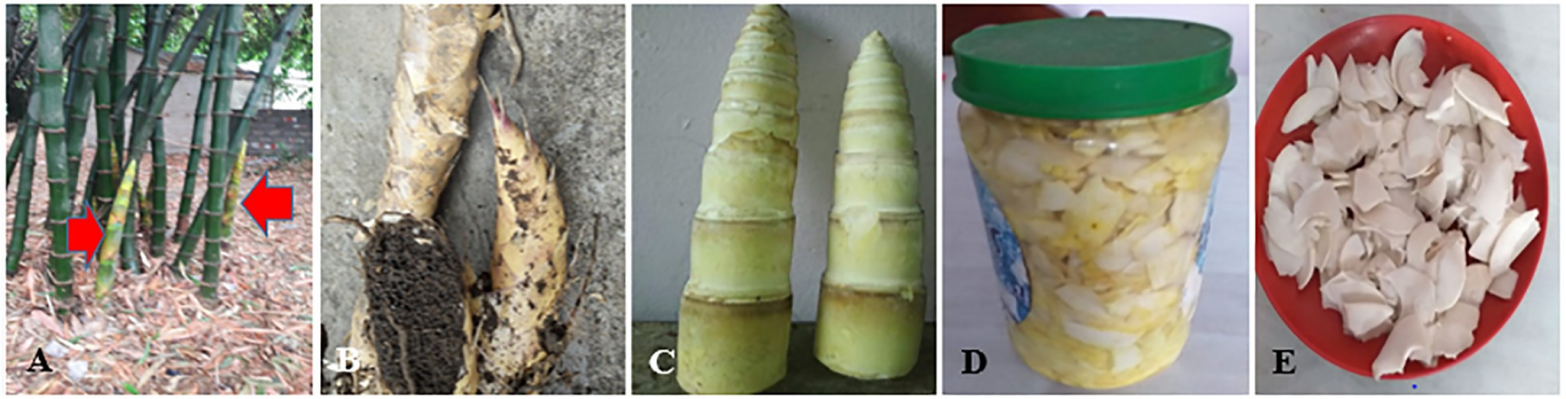
The bamboo used as food: A- Young bamboo shoot (*B*. *balcooa* shown with arrows); B- Sprouting shoot of *C*. *latifolium*. C- Young culms (*B*. *nepalensis*) ready to be chopped; D- Chopped bamboo culms canned for preparing pickle; E- Young culms chopped for fresh vegetables.

Young culms (locally known as tama; Fig 7E) of *B*. *nepalensis*, *B*. *nutans* subsp. *cupulata* and *B*. *tulda* are used for skin rejuvenation and reducing blood sugar;leaf paste of *B*. *bambos* is used for curing snake and scorpion bites;young culm of *B*. *nepalensis*, *B*. *tulda*, *B*. *bambos* and *B*. *balcooa* are useful for maintaining hypertension and weight loss, and sub-branches of *B*. *nutans* subsp. *cupulata* are used as toothbrush because they reduce toothache during oral infection.

The level of informant agreement was high for most of the use categories (mean ICF = 0.92), and a total consensus was observed in the use of bamboo against snake and scorpion bites, while it was the lowest level (0.81) for the application of bamboo in reducing weight loss (Table 8).

**Table 8:** List of bamboo species used for medicinal purposes and informant consensus factors.

Bamboo species	Use categories	No of taxa (N_t_)	No of use reports (N_ur_)	ICF
*D*.*strictus*, *B*. *tulda* and *B*. *nutans* subsp. *cupulata*	Fresh cut or laceration and wounds	3	21	0.90
*B*. *nutans* subsp. *cupulata* and *B*. *tulda*	Nocturnal enuresis	2	32	0.96
*B*. *nutans*, subsp. *cupulata* and *B*. *tulda*	Jaundice	2	65	0.98
*B*. *balcooa*, *B*. *tulda* and *B*. *nutans* subsp. *cupulata*	Sores	3	32	0.93
*D*. *strictus* and *B*. *nepalensis*	Cough and respiratory disorder	2	17	0.93
*B*. *nepalensis*, *B*. *tulda* and *B*. *nutans* subsp. *cupulata*	Skin rejuvenation	3	15	0.85
*B*. *nepalensis*, *B*. *tulda* and *B*. *nutans* subsp. *cupulata*	Diabetes	3	26	0.92
*B*. *nepalensis*, *B*. *balcooa* and *B*. *tulda*	Hypertension	3	43	0.95
*B*. *nepalensis*, *B*. *balcooa* and *B*. *tulda*	Weight loss	3	12	0.81
*B*. *nutans* subsp. *cupulata* and *B*. *nepalensis*	Oral infection	2	56	0.98
*B*. *bambos*	Snake and scorpion bite*	1	23	1.00

### Bamboo multipurpose uses as food, fodder and fuel

**Fig 8:**
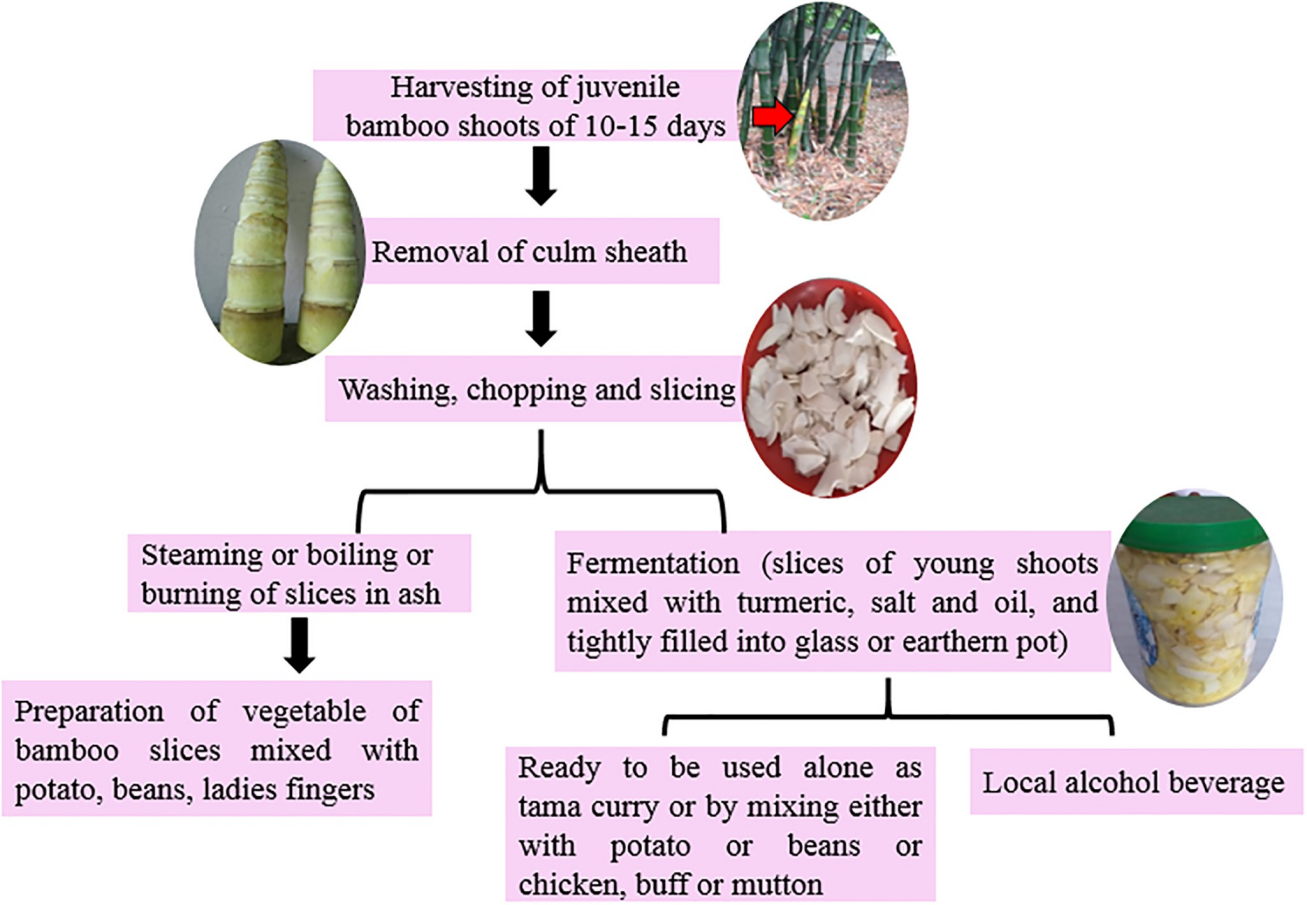
Consumption pattern of juvenile bamboo shoots in the study area.

Since ancient times, bamboo have been used as food for human beings and fodder for livestock,considering the high nutritional value of young culms and their leaves. In our study sites, the young culm (*tama*) of certain species of bamboo (*B*. *nepalensis*, *B*. *balcooa* and *B*. *nutans* subsp. *cupulata*) have been used as food for human consumption. A major vegetable is prepared from the freshly cut young shoots. The bamboo shoot slices are usually mixed with other vegetables such as potato, broad bean and lady’s finger. The fermented bamboo shoots (Fig 7D) are used to prepare curry, soup, pickle, condiment (*chatni*), and the *Majhi* people prepare traditional curry of young culm as well as fermented slices mixed with pork, buff, chicken, fish and egg which is famous as tama curry (Fig 7A–7E). The sheaths of young bamboo shoots are removed, the white part is cleaned, cut into thin slices and boiled in water with small amounts of wooden ash, rinsed with clean cold water to remove bitter content, sun-dried on bamboo woven baskets (*supli*), and mixed with oil, turmeric powder and salt (Fig 8). It is then placed in air-tight containers in a dry place mostly in sunny area, to fasten the fermentation rate. Boiled and dried fermented bamboo shoots can be stored for two years. The culm, rhizome, sheath and branches of naturally dead or broken or distorted bamboo are used as firewood.

### Bamboo multipurpose uses as construction materials

**Fig 9:**
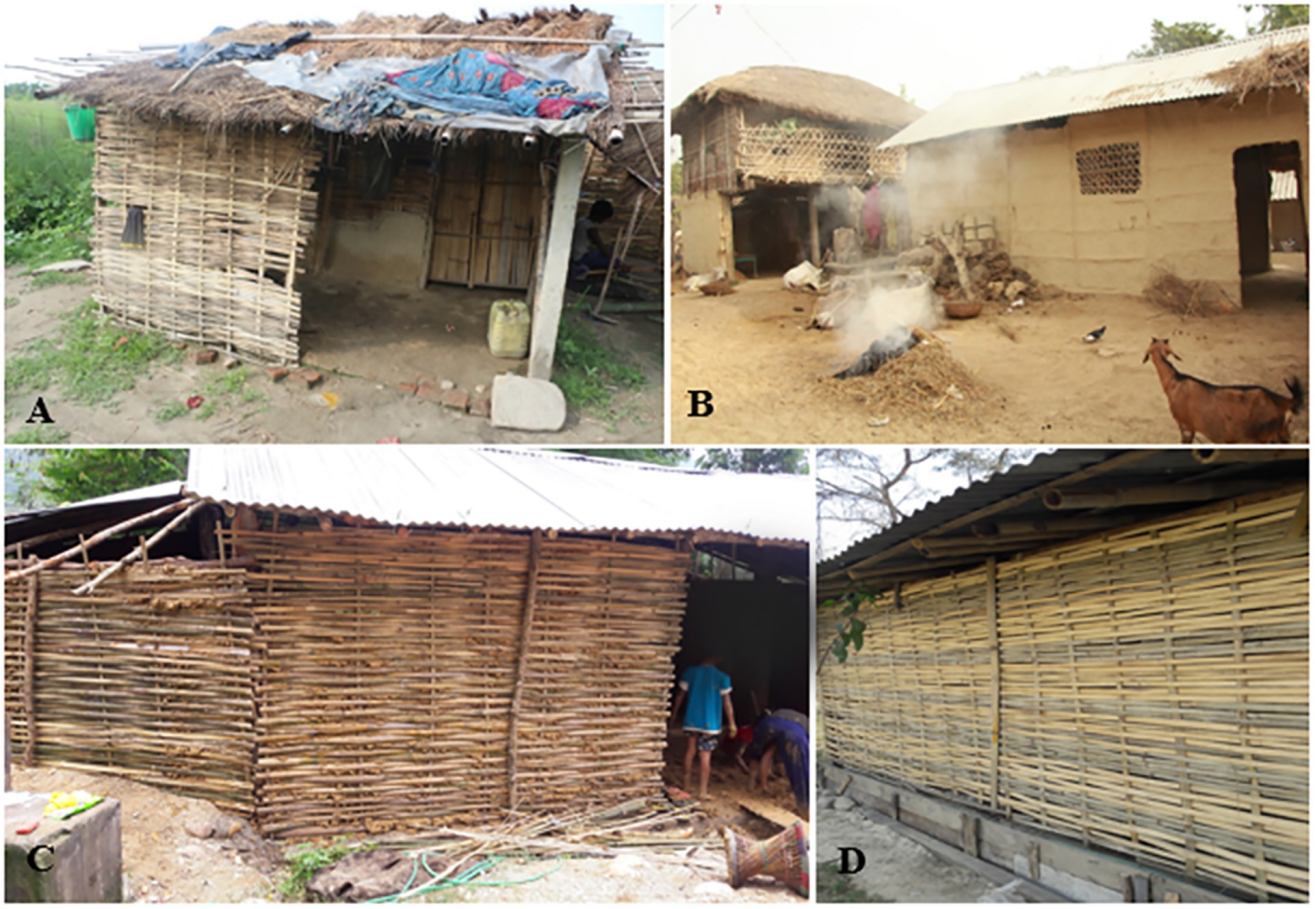
Bamboo used for the construction of thatched houses.

From KII and FGD, it has been understood that bamboo are common sustainable resources for construction materials. In deprived communities, bamboo culms have been used for home materials such as wall, roof, flooring, door, window (*jyaphri*), and poles (Fig 9). In some cases, the local people use bamboo for making narrow cross bridges over the brooks. Mature, straight, thick and sturdy bamboo culms (*B*. *tulda*, *B*. *balcooa*, *B*. *bambos*, *B*. *nepalensis* and *B*. *nutans* subsp. *cupulata*) generally of more than seven years old were used as construction materials or the main pillars of traditional houses. *Bambusa tulda* is the most prioritized -grown bamboo species widely used in traditional household construction and other local uses. Thin bamboo with long internodes (*B*. *nutans* subsp. *cupulata*) is used to make a roof frame, wall, and floor mat.

### Bamboo multipurpose uses in various agricultural purposes

**Fig 10:**
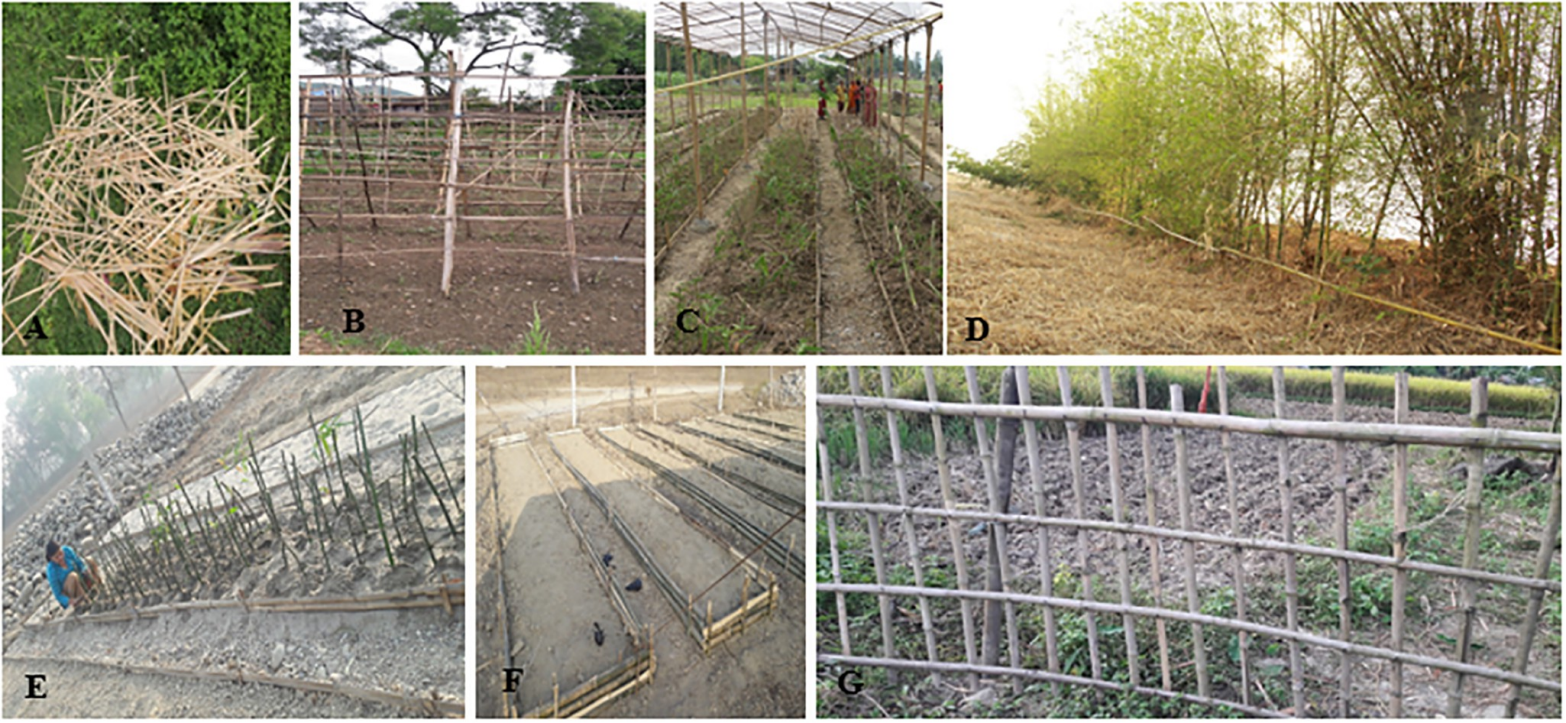
Application of bamboo for agricultural purpose: A- Bamboo splits; B- Bamboo for climbers; C- Bamboo for preparation of nursery shed; D- Bamboo used as live fencing to prevent entry of unwanted domestic and wild animals; E and F- Bamboo in preparation of nursery for bamboo seedlings; G- Bamboo fencing around paddy field.

During the household survey and participant observation, we found that three bamboo species are used for agricultural purposes. These included the construction of the greenhouse for vegetable farming (Fig 10A–10E), preparation of *bei* (circular fencing around the plant), poultry farms, pig farms, fencing and support for climbing and creeping agricultural and ornamental plants. Four to seven years old culm of *B*. *tulda*, *B*. *balcooa* and *B*. *bambos* are used to make handle of axe, plough, cart, hoe, spade, trowel, sickle, knife and sword. Destruction of crop and disturbance of the agro-ecosystem is a rampant problem in the study area. The privileged and underprivileged communities solve these problems by establishing bio-fencing (Fig 10D). Accordingly, they plant various bamboo species (*B*. *bambos*, *B*. *tulda* and *D*. *strictus*) in close spacing or fencing around the boundary of cultivated fields using dead bamboo culms so as to minimize or avoid the entrance of domestic and wild herbivores.

### Bamboo multipurpose uses as artifacts

**Fig 11:**
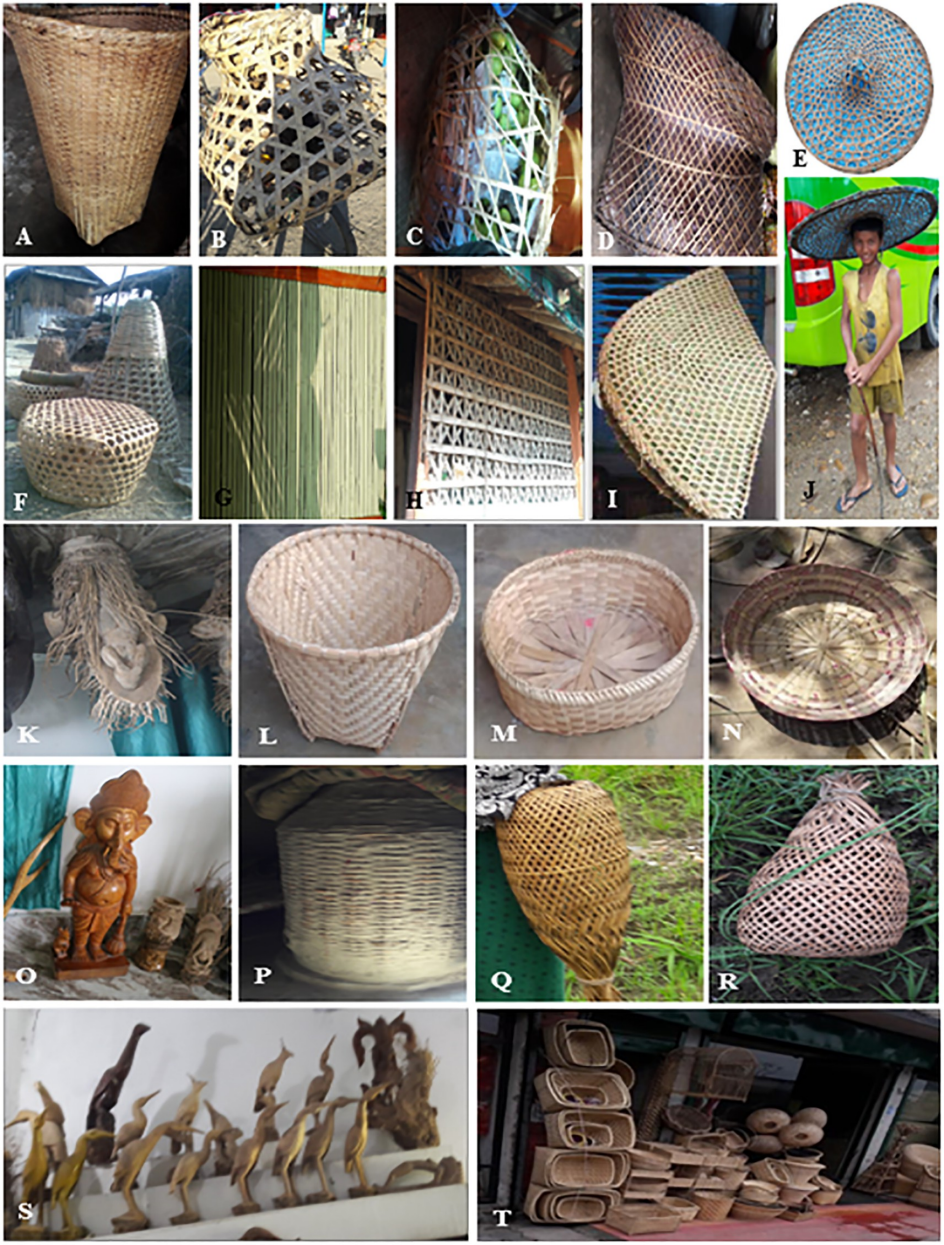
Bamboo artifacts: A- *Thunche*, B- *Tokari* being used to transport poultry, C- *Tokari* being used to pack mango, D-Rectangular *ghum*, E- Circular *ghum* with central hood to fit with the head of the user, F- *Aakhne doko* and basket used to cover chickens, G- Bamboo wall, H- *Jyaphri*, I- Semicircular *ghum*, J- A boy wearing a circular *ghum*, K- A bamboo rhizome being carved into an artifact, L- *Dalo*, M- *Tokari*, N- *Dhakiya*, O- An idol of Lord Ganesh, P- *Bhakari*, Q- *Gidra*, R- *Torungo*, S- Birds carved from bamboo rhizomes, T- Bamboo artifacts kept for sell in local market.

Bamboo artifacts and *mandro* (Woven mats) are traditional products in *Dome*, *Majhi* and *Mushahar* communities of our study sites. They have adopted such skills since time immemorial. These diverse products have become an indispensable part of the daily life of deprived communities. There are nearly 26 categories of bamboo artifacts (Table 9) including *doko/thunche* (conical woven basket used to carry grass, stones, bricks, etc.), *dhakiya* (small basket), *thal* (plate), *bansuri* (flute), *gamala* (vase), *bharyang* (ladder), *khat* (scaffolding), *supli/supo* (big round plate), *dali/dalo* (small to large sized bamboo pot to keep grains), *torungo* (fish keeping basket used by fish vendors), *makhara* (face cover for oxen not allow them to graze while ploughing), *bhakari* (large woven basket up to the breast height to place harvested crops), *dori* (rope made from spiral coiling of bamboo splits), *chalno* (sieve), *gidra* (fish collecting pot during fishing), *khamba* (bamboo poles), *hate pankha* (hand fan), *dhungro/chungye* (bamboo pot for liquid materials mainly ghee and oil), *ghum* (Nepali umbrella) (Fig 11A–11T). These products can be made from 1–4 years old culms of *B*. *nutans* subsp. *cupulata*. Bamboo pot *ghiri*, *dhungro/chungya* are prepared from 4–7 years old culm of *B*. *tulda*and *B*. *balcooa* especially for keeping milk and oil respectively. Similarly, *kurchi/mech* (chairs) are prepared from 3–7 years old culm of *D*. *strictus* and *mudha* (stools) are prepared from 3–7 years old culm of *A*. *patellaris* and *C*. *latifolium*.

**Table 9:** List of bamboo artifacts used in the study areas (number of respondents, n = 30 per site).

Vernacular name	English name	Number of respondents in each district							Average number
		UDH	SPT	SN	DHA	MAK	SAS	SIR	
*Doko/Thunche*	Basket	7	12	10	11	15	19	18	13.14±1.65
*Dhakiya*	Round basket	10	8	7	12	13	18	12	11.42±1.37
*Tokari*	Small basket	7	4	8	14	19	10	13	10.71±1.89
*Mandro/Chatahi*	Mat	15	13	19	19	21	20	24	18.71±1.39
*Bansuri*	Flute	6	3	4	3	8	12	7	6.14±1.22
*Gamala*	Vase	2	3	4	12	17	23	17	11.14±3.12
*Bharyang/Lisno*	Scaffolding/ladder	25	24	25	24	19	22	25	23.42±0.84
*Hate pankha*	Hand fan	2	7	9	10	12	12	8	8.57±1.30
*Bei*	Tree guard	25	24	24	25	25	25	25	24.71±0.18
*Ghum*	Nepali umbrella	2	1	2	1	3	2	1	1.714±0.28
*Bhakari*	Granary(Large basket)	23	22	25	25	22	23	19	22.71±0.77
*Dhanus*	Bow	1	2	1	1	2	1	2	1.42±0.20
*Dori*	Ropes	25	25	25	24	23	22	19	23.28±0.83
*Makhara/Makaura*	Bull mask	1	4	6	8	7	6	9	5.85±1.01
*Ghiri*	Bamboo milk pot	0	1	1	3	7	2	1	2.14±0.88
*Dhungro/Chungye*	Bamboo oil pot	0	0	0	1	9	7	0	2.42±1.46
*Chalno*	Sieve	19	22	24	23	18	15	9	18.57±1.98
*Naglo/Supli*	Winning receptacle	25	25	25	25	25	21	19	23.57±0.94
*Jhyaphri*	Window	0	0	2	4	12	2	0	2.85±1.62
*Khamba*	Building poles	25	25	25	25	25	20	25	24.28±0.71
*Gidra*	Fish collecting jar	1	0	0	0	0	2	15	2.57±2.01
*Torungo*	Fish collecting jar	0	0	0	0	0	1	13	2±1.8
*Phohorrakhnebhando*	Dust bean	7	9	12	10	7	9	8	8.85±0.67
*Kurchi*	Chair	3	0	0	0	12	5	0	2.85±1.69
*Mech*	Table	3	10	9	5	12	19	16	10.57±2.14
*Dala*	Medium size basket	22	24	21	17	23	25	25	22.42±1.06
*Tokari*	Round basket	26	28	22	21	27	29	19	24.57±0.08

UDH: Udaypur,Hadiya, SPT: Saptari,Tikulia, SN: Siraha,Naudega,DHA: Dhanusa, Aauribaba, MAK: Mahottori,Kantibazar,SAS: Sarlahi,Sasapur, SIR: Sindhuli Ranibans.

### Bamboo usesin religious ceremonies and for ornamental purpose

**Fig 12:**
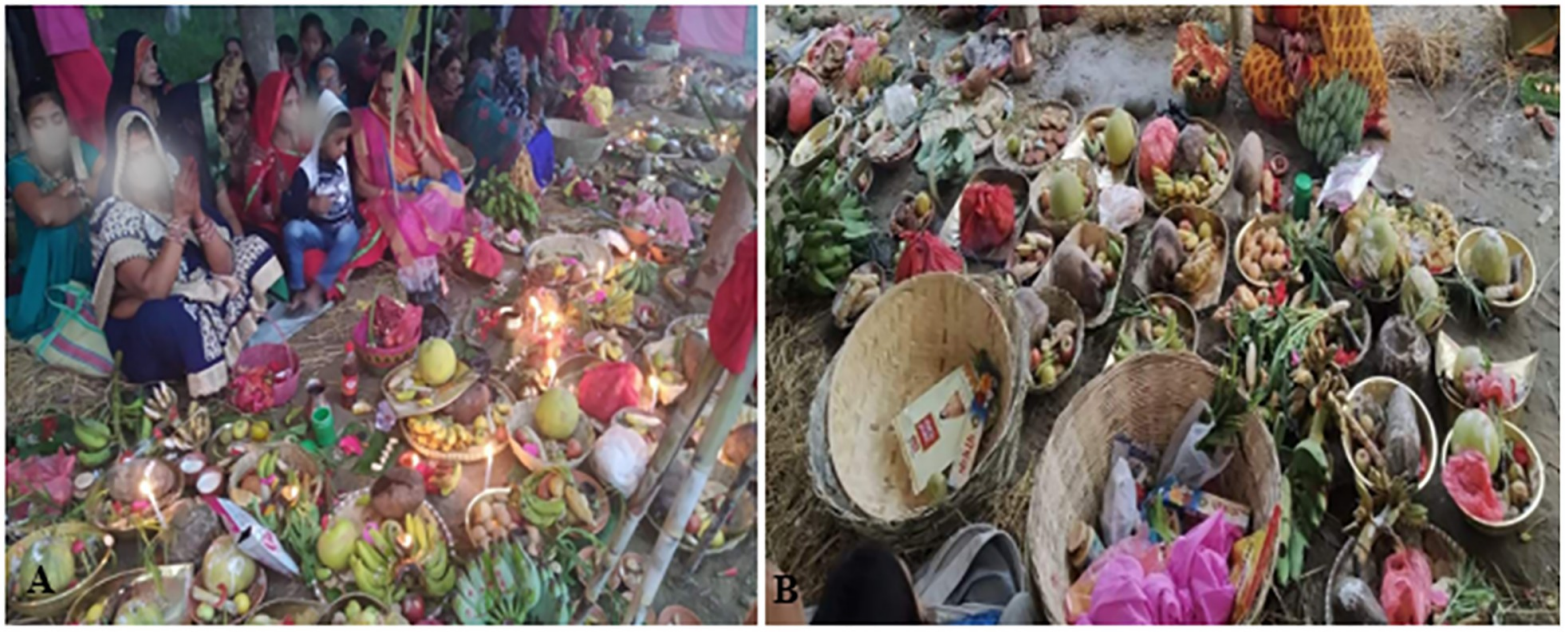
A-B. Local people using various bamboo artifacts during religious ceremony (Chhath pooja).

The inhabitants of the study sites have diverse cultural practices. Altogether, four bamboo species (*B*. *vulgaris*, *B*. *nepalensis*, *B*. *nutans* subsp. *cupulata* and *C*. *latifolium*) are used for religious purposes. These species are used in different cultural and religious ceremonies like *nwaran* (baptism), *bartabandha*/*upanayan* (ceremony of wearing sacred thread), *bibaha* (marriage), *mritu* (death) and different worshipping ceremonies. Culm of *B*. *vulgaris*, *C*. *latifolium* and *B*. *nutans* subsp. *cupulata* are used for making *mandap* (tent house), *swagatdwar* (welcome gate) during the marriage, *bartabandha* and other religious ceremonies like *saptaha* (worship and recitation of religious books like *mahabharat*, *purans*, etc., for seven consecutive days organized by an individual or family or community level), *kulpooja* (worship to the ancestors), and *mandap* in and nearby water bodies during *chhat pooja* (worship of Sun as God; Fig 12A and 12B). Bamboo artifacts like *dalo*, *dhakiya*, *supli* are used to decorate, transport and offering fruits and other worship items during *chhat* festival. *Bansuri* (flute) is made from 1–3 years old culm of *C*. *latifolium*. Coffins are generally made from culm of *B*. *nutans* subsp. *cupulata*, *B*. *tulda* and *B*. *balcooa*. Besides these, *B*. *vulgaris* is planted in house yards forornamental purposes.

### Bamboo uses as river embankment and othere cological purpose

**Fig 13:**
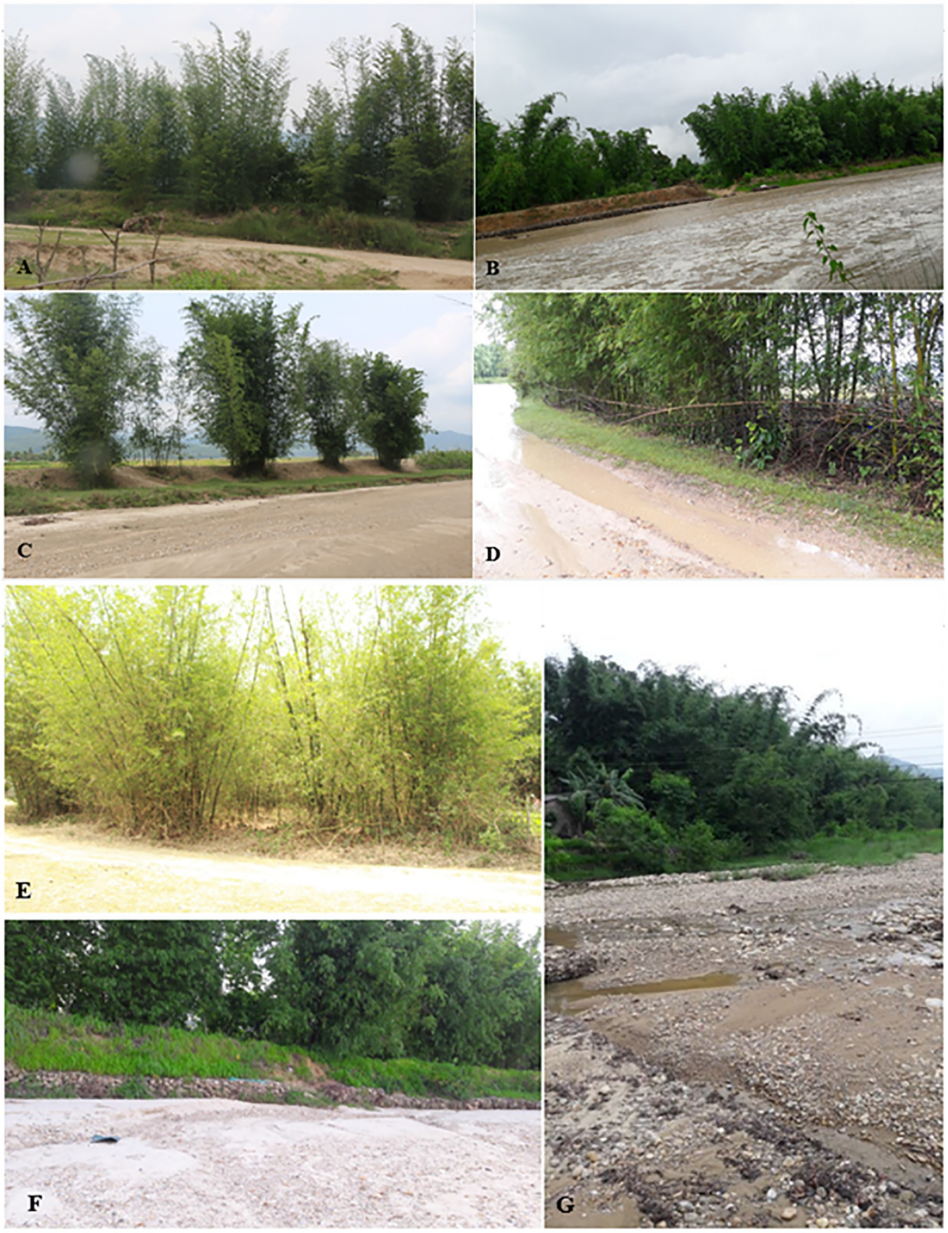
Bamboo used for embankment: A, B, C and D- *Bambusa tulda*; E- *B*. *balcooa*; F and G- *B*. *nutans* subsp. *cupulata*.

**Fig 14:**
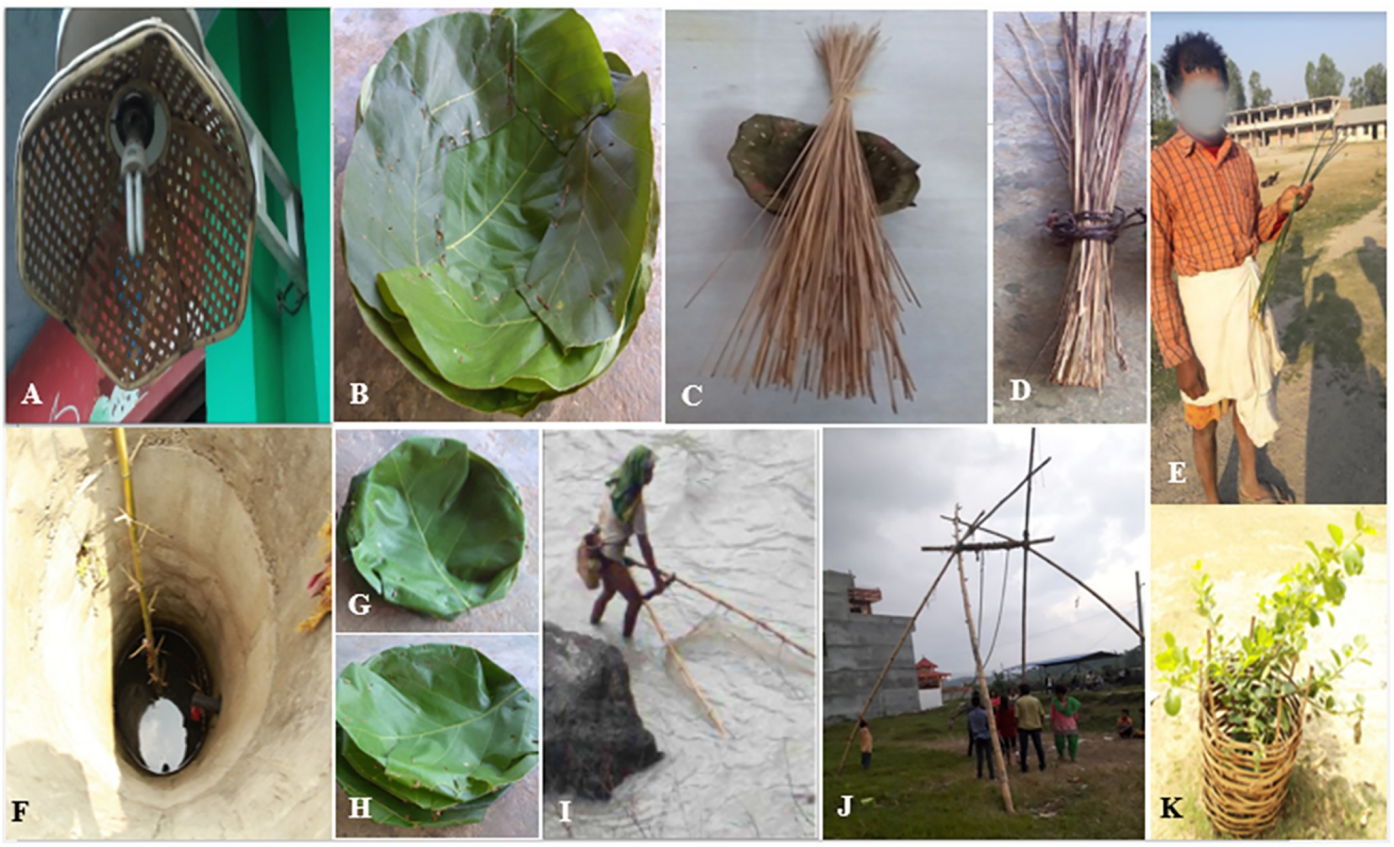
Miscellaneous uses of bamboo: A- Lamp shed, B- *Tapari* (large plate made of *Shorea robusta* leaves stitched with thin bamboo splits (*sinka*) of *C*. *latifolium*, C- *Sinka* and *duna*, D- *Bhutuna* prepared from mature *C*. *latifolium* splits, E- A man carrying sub-branches of *B*. *nutans* subsp. *cupulata* for making tooth brush, F- Culm of mature (6 years old) *B*. *tulda* being used as a ladder to reach the bottom of well, G- *Bauta*, H- *Duna*, I- Bamboo culm of *C*. *latifolium* being used as fishing rod, J- Mature culms (6 years old) of *B*. *tulda* used in swing (*ping*) during dashain festival, K- *Bei* for plant protection.

Three species of bamboo (*B*. *balcooa*, *B*. *tulda* and *B*. *nutans* subsp. *cupulata*) are grown by the privileged and underprivileged communities for the establishment of the river embankment (Fig 13A–13G) in field bunds, bench terraces, marginal lands, fallow lands, stream banks, seasonal ravines and riverbanks. About 2–3 years old culms of *C*. *latifolium* and *D*. *strictus*areused for making flag poles. The dried culms of *C*. *latifolium* and *B*. *nutans* subsp. *cupulata* are used to prepare *sinka* (very thin splits of about 20–30 cm in length) to stitch the leaves of *sakhuwa*/*sal* (*Shorea robusta*), and other large leaves to prepare *duna* and *tapari* (smaller and larger leaf plates respectively; Fig 14B, 14G and 14H). These *duna* and *tapari* are most commonly used during religious ceremonies to place worship items and even for serving food at home and *Nepali chulo* (a traditional Nepali restaurant). Almost all the bamboo species recorded in the field are also used to make *bhutuna* (almost cylindrical shaped bamboo splits about one foot in length arranged in a bundle), which are common in the kitchen of the local people who use it to stir while frying corn, beans, or wheat and other cereals. We also observed 3–5 years old culm of *C*. *latifolium* also being used for fishing (Fig 14I).

### Market problem

**Fig 15:**
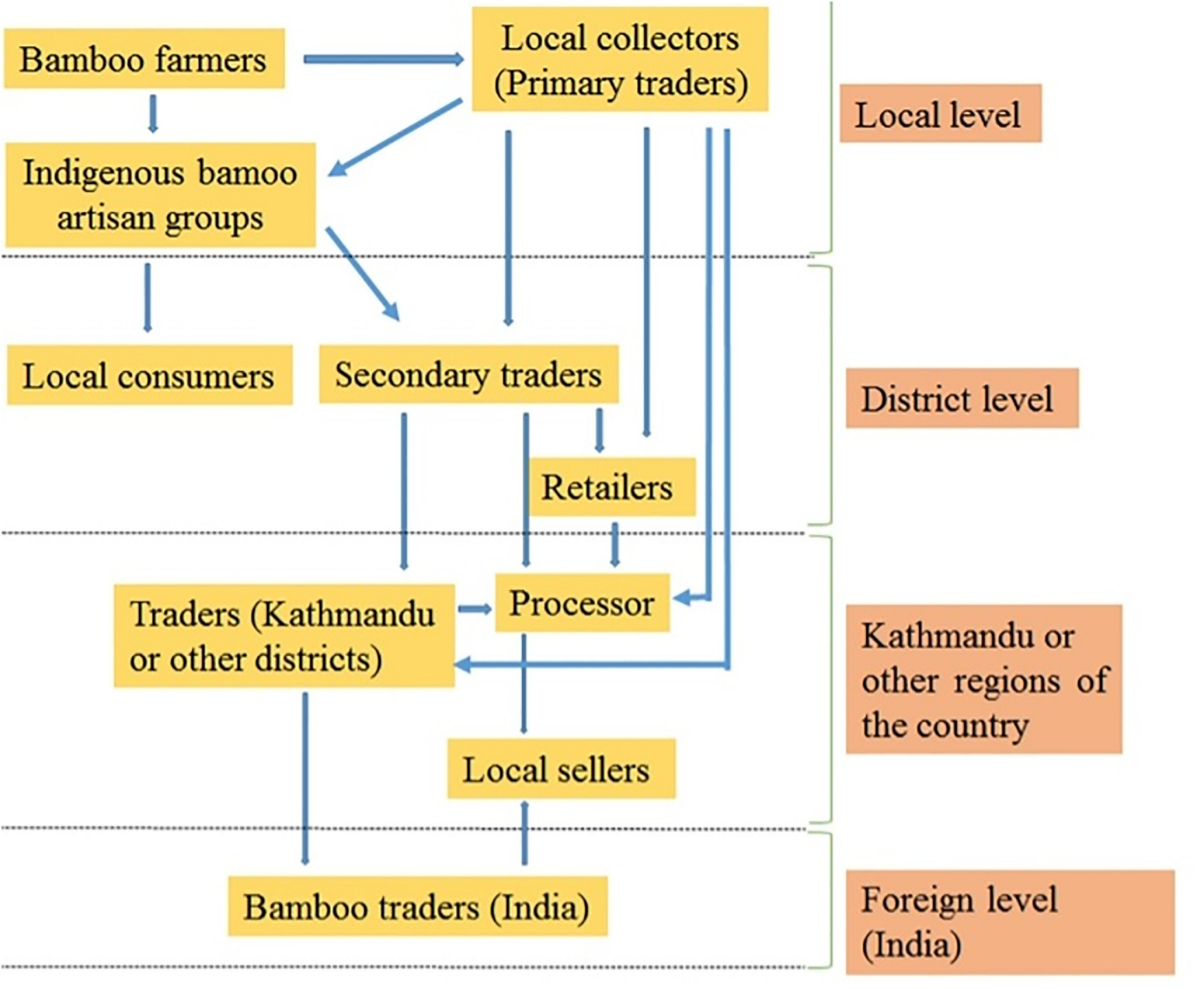
Trade channel of bamboo and bamboo artifacts.

The study sites do not have the government-owned firms or private agencies to collect harvested bamboo culms and artifacts. The bamboo farmers and indigenous bamboo artisan groups were not able to obtain the proper benefit from bamboo artifacts marketing due to the intervention by brokers, middlemen and traders. The lack of bamboo entrepreneurship in marketing also reduces the margin of profit for bamboo farmers as well as local indigenous bamboo artifact groups. Some noteworthy market-related issues in the study areas are variable prices within the district due to limited value chains, fluctuation in the supply and demand, lack of information about the actual selling price of the bamboo culm to the farmers, lack of storage facilities for bamboo culms and bamboo made artifacts (Fig 15). Increased use of easily available plastic products in the market implies a decline in the use of traditional bamboo artifacts.

## Discussion

Bamboo user groups (BUGs) were identified through focus group discussion (FGD), which is considered an appropriate tool for assessing people’s perceptions of a specific area of interest. To address our objectives and conveniently segregate data, BUGs were grouped into different strata (priviledged, underpriviledged and deprived). Bamboo shoots popularity is increasing in Asia and other markets around the world [57]. We focused on variations of lore among eighteen ethnic and three bamboo artisan communities. The results provided notable information regarding the livelihood pattern, utilization aspect of bamboo, bamboo plantation, and lore of individuals in the application of bamboo resources for various purposes and market trends. Many households of deprived communities, especially bamboo artisan groups of the study sites depend upon bamboo to meet their daily requirements. Bamboos play a big part on their alternatives in livelihood options, although there has been a shortage of bamboo culms in the study sites.

### Bamboo diversity and their utilization pattern

Traditional utilization pattern of various bamboo species for diverse purposes by the local people directly or indirectly contributes to the diversity of bamboo species and their management [14]. During this research, we found that the majority of the species of bamboo (*A*. *patellaris*, *C*. *latifolium*, *B*. *nutans* subsp. *cupulata*, and *B*. *nepalensis*) are used for livestock fodder. Bamboos serve as a better alternative as supplement fodder because their leaves contain rich amounts of carbohydratesin contrast to other fodders [58].

In most Asian countries and America, fresh bamboo shoot is a trendy edible vegetable [59]. In our study sites, succulent young culm of bamboo (*B*. *nepalensis*, *B*. *balcooa* and *B*. *nutans* subsp. *cupulata*, *C*. *latifolium*) are mostly used as fresh vegetables in various ethnic communities. A similar practice is documented in other studies. Tamang and Tamang [60] reported that *tama* is popular as a non-fermented vegetable curry among the ethnic group of Sikkim, India. In other parts of Nepal, bamboo shoots are fermented with turmeric and oil, and cooked with potatoes to organize an item called *aalu tama*. Our study also showed that *Majhi* community prepared local beverages from the fermented bamboo young shoot (commonly referred to as *sandeko tama*). It is similar to Chinese practice where the sap of young stalks is tapped during winter to make beverages [61].

The young bamboo shoots are enriched with a larger quantity ofmacronutrients like amino acids, proteins, carbohydrates, fat, and fiber compared to the fermented and canned shoots. The mineral content, namely calcium, potassium, iron, phosphorus and vitamin E,was highest in raw shoots, followed by fermented shoots. Thus, as a recommendation for food consumption, freshly harvested shoots are preferred rather than fermented shoots due to their richer nutritional content [24].

Branches and leaves of young bamboo shoots are also widely used in traditional medicines. Of the nine species of bamboo, six species were found to be used for traditional medicines (Table 7). Like in other rural communities of Nepal [62–69], the knowledge about traditional uses of bamboo and other medicinal plants in our study area is inherited from the seniors and other elders to the juniors. In many cases, such knowledge and skills are continuously transferredverbally from generation to generation; however, it remains confined to a limited group of people [70]. The traditional knowledge about medicinal plants is rapidly diminishing [71] as young generations are found paying less interest towards the traditional medicinal utilization of plants [72]. Parajuli [73] has underlined the utmost importance sharing and documenting the knowledge and experiences of the local people for traditional uses of plants which can contribute to economic development and genetic resource conservation.

Among the wide and diverse applications of bamboo, the artifact is one of the common scopes of bamboo application. Our study recorded 27 different artifacts application in the Central Siwalik region. A previous study reported only 14 major uses, namely containers, pillars for shelter, drying racks, roof beams or trusses, thatch supporting lattices, fences, posts and so on in eastern Nepal [20]. Comparatively, the limiteduses in eastern Nepal might be due to the inclusion of a higher sample of privileged communities and a lack of study in artifact-specific ethnic groups. Our study, on the other hand, targeted the ethnic groups *Mushahar*, *Dome* and *Majhi*, who still primarily rely on the bamboo artifacts for their livelihood. Traditionally, bamboo artifacts were made by individual households in the beginning, and the primary purpose of bamboo artifacts was to achieve self-sufficiency. However, during our field survey, we found that privileged and under privileged people were considerably less involved in making bamboo artifacts, which could be due to the increased use of plastic-made materials. Only deprived communities were involved for making bamboo artifacts.

During FGD and field observation, we found that *B*.*nutans* sub sp. *cupulata*, *B*. *vulgaris* and *C*. *latifolium* have higher cultural reverence in festivities like *Chhath pooja*, marriage and other traditional rituals for which people have been using bamboo for thousands of years. Traditional bamboo artifacts in our study sites usually possess rich cultural properties and extensive marketing prospects. We found that the development of traditional bamboo artifacts can be combined well with cultural inheritance and fulfilling the livelihood requirements. The long-lasting foundation of cultural significance of the species can inherently prevent the depletionof the bamboo in the area. However, adaptation to the use of modern products may threaten the preservation of species for the cultural uses [74–76].

In our study sites, *B*. *balcooa*, *B*. *tulda* and *D*. *strictus* have been used for the rehabilitation of degraded land as well as eco-restoration of seasonal ravines and riverine areas. The result is comparable to the study in the ecologically fragile land of northeastern hilly region of India, where *B*. *nutans*, *B*. *arundinacea* and *D*. *hamiltonii* have been usedfor eco-restoration of Jhum fallows [77]. Similarly, *B*. *bambos*, *B*. *nutans*, *B*. *pallida*, *D*. *strictus*, *D*. *hamiltonii*, *D*. *longispathus*, *D*. *hookeri*, *D*. *sikkimensis*, *Melocanna baccifera*, *Chimonabambusa callosa*, *Schiostachyum polymorphum*, *Neomicroaclamus mannii*, *Gigantochloa andamanica*, *Sinarundinaria falcate* have been found to be of great potential and can effectively be used for the restoration of the degraded areas of the Meghalaya region of India [78].

### Plantation development and market trends of bamboo

During FGDs, key informant interview and direct observation, we noted that adequate knowledge ofbamboo plantation, management, and marketing were lacking amongst the local bamboo users. This finding is in agreement with Das [29], who reported that the bamboo farmers, artisan groups and traders do not have sufficient information on the technical aspectsof bamboo plantation and marketing. We also found that there is also scarcity of planting stocks for large-scale bamboo plantations in homesteads, farmlands, and fallow lands, as well as in seasonal ravines and riverine areas. Non-availability of planting stock of bamboo is the major constraint for bamboo planting because it is traditionally propagated by rhizome cutting, which is very tiresome and time-consuming, on top of that, bamboo seeds are not easily available as almost all the bamboo species flower after a long interval [79].

The most commercially marketed shoots for vegetable purposes in our study sites are *B*.*tulda*,*B*.*balcooa*, *B*.*nepalensis* and *B*. *nutans* sub sp.*cupulata*. In India, *B*. *balcooa*, *B*. *bambos*, *B*. *tulda*, *Chimonobambusa callosa*, *C*. *hookeriana*, *Dendrocalamus flagellifer*, *D*. *giganteus*, *D*. *hamiltonii*, *D*. *hookeri*, and *Melocanna baccifera* are mostly used for food [24]. Over the duration of field visit, the current price ofone mature bamboo culm (mainly *B*. *tulda*, *B*. *balcooa*, *B*. *nutans* subsp. *cupulata* and *D*. *strictus*) increased from NRs 150 to 370 (equivalent to 1.26 to 3.12 US$) and young shoot (*tama*) ranges from NRs 40 to 60 (equivalent to 0.336 to0.51 US$). Our result is supported by Jha and Yadav [32], who reported that the price of bamboo culms increasing day by day in Rautahat district, Nepal. This sudden price hike suggests the scarcity of the products and the risk of unavailability followed by disappearance of the products from local communities. The survey of trade centers showed that *B*. *nutans* subsp. *cupulata*, *B*. *tulda*, *B*. *balcooa* and *D*. *strictus* have a huge potential to enhance the livelihood and socioeconomic development by manufacturing the artifacts that could be easily sold. After consultation with the local people, district forest office, traders and community development organizations, these species were prioritized because of their potential commercial values. However, local people were mostly unaware of the scientific plantation of thesebamboo, and the income generation through the commercialization of such species was negligible. This is one of the instances ofthe worrying trend in the consumption of bamboo, and hence, it underpins the need for plantation, conservation and management of bamboo in our study site.

### Bio-efficacy, novelty and future perspectives of bamboo

The comparison of local uses and ethnomedicinal properties of six bamboo species reported from our study area showed that traditional use was coherent with known pharmacological properties. Our studies highlight the importance of bamboo in local diets and medicine but also indicate that the current trends in the harvesting of *B*. *tulda and B*. *nepalensis* may not be sustainable and could affect species availability in the future. The medicinal uses of bamboo by ethnic community of Central Siwalik region of Nepal needed to be reinforced with phytochemical studies in order to authenticate their bio-efficacy. This matter was raised in multiple ethnomedicinal studies, but only a few provided the required evidences [80].

The present study is the first documentation on ethnobotanical uses of nine bamboo species used by the inhabitants of Central Siwalik region of Nepal. The current ethnobotanical uses of reported bamboo species were compared with previous studies conducted in Siwalik region and other areas of Nepal [4, 21, 27, 31, 32, 44, 81] to find the novelty. There is scanty of reports on medicinal uses of *B*. *balcooa*, *B*. *bambos*, *B*. *nepalensis*, *D*. *strictus* and *B*. *nutan*subsp.*cupulata* in context of Nepal. The data collected from the study area revealed considerable differences in the plant parts and their utilization as reported from other regions. Some newly documented bamboo species used in medicinal purposes include *B*. *balcooa*, *B*. *bambos*, *B*. *neplensis*, *D*. *strictus* and *B*. *uutans* subsp. *cupulata*. These bamboo species with new medicinal uses could be studied further to screen bioactive compounds and their pharmacological activities. Bamboos are alternative source of forest and they have great role in conservation biology and become priority concerns. With knowledge of the awareness of the conservation biology and environment however people have to fulfill the enormous demands of the markets, they have to exploit the limited resources.

## Conclusion

We have documented nine bamboo species which have potential ethnobotanical importance. Our study suggested that the bamboo species can be considered as valuable non-timber forest products which contribute significant socioeconomic impact on the livelihood of the local people. Most of the bamboo species were used for agricultural applications. However, deprived people are often away from the reach of the benefits of bamboo resources. Plantation of various species of bamboo needs to be carried out with public participation, and the establishment of bamboo refinery industry is a current demand so as to economically empower the deprived communities. The use of bamboo by deprived communities fairly influences the fulfillment of everyday necessities to the community. However, until their artifacts are considered cheap and low-quality items, the chances of improving the economic status of the bamboo artisan groups is impossible.

There is a lack of information on marketing and bamboo’s demand and supply trends. Therefore, further detailed marketing appraisal is required. There is a need to develop new livelihood opportunities through bamboo plantation and establishment of bamboo industries by improving product quality and targeting high-value markets. At the same time, it is equally important for the sustainable management of existing bamboo species. Propagation of high-priority bamboo species, as mentioned in this study, in degraded and fallow land with technical assistance from the local government would be helpful to facilitate the maximum uses of bamboo, which directly augments the livelihood pattern of deprived communities. This study revealed the importance of bamboo in the traditional health care system. Pharmacological studies of traditionally used bamboo are thus an important line of research to pursue.

## Supporting information

S1 File Inclusivity in global research. (DOCX)

S1 Table (DOCX)

S2 Table (XLSX)

S1 Fig (XLSX)
