# Supplementary material for: Traditional utilization of bamboo in the Central Siwalik region, Nepal
Source: PLoS One. 2024 Jan 30;19(1):e0296886. doi: 10.1371/journal.pone.0296886 (PMC10826958; doi:10.1371/journal.pone.0296886)
Supplement: S1 Table — (DOCX) [file pone.0296886.s002.docx]

**Supplementary file 1**

**Questionnaire survey**

1. Name of the village:
2. Area/Ward number:
3. Ecological zone:
4. Household number:
5. Name of the respondent:
6. Cast/Ethnicity: i. Indigenous ii. Immigrated (Since when?)
7. Gender:
8. Occupation:

| Type of occupation | Male | Female | Annual income | Source of income |
| --- | --- | --- | --- | --- |
| Farmer |  |  |  |  |
| Bamboo business |  |  |  |  |
| Others |  |  |  |  |
| Unemployed |  |  |  |  |
| Employed |  |  |  |  |
| Government employed |  |  |  |  |
| Self employed |  |  |  |  |
| Daily wages |  |  |  |  |

1. Family size:
2. Age:
3. Maritalstatus:
4. Education status:
5. Illiterate ii. Literate iii. Educated (Primary, Secondary, University level).
6. Land holding:

| Land type | Khet* | Bari** | Marginal land | Private forest | Total |
| --- | --- | --- | --- | --- | --- |
| Bigha/Kattha/Dhur |  |  |  |  |  |
|  |  |  |  |  |  |

*Field which are irrigable for rice cultivation (popularly known as paddy field)

** Non-irrigable land used for crop production other than rice.

1. Food sufficiency:

| For how long (Months) | Before 10 Years | Now |
| --- | --- | --- |
| <1 |  |  |
| 2 |  |  |
| 4 |  |  |
| 6 |  |  |
| 8 |  |  |
| 10 |  |  |
| 12 |  |  |

1. Livestock farming:

| Types | Number | Farming practice | |
| --- | --- | --- | --- |
|  |  | Stall feeding | Grazing |
| Buffalo |  |  |  |
| Cow |  |  |  |
| Goat |  |  |  |
| Others |  |  |  |

1. Have you planted bamboo? Yes/No
2. If yes, in what type of land?

i. Khet ii. Bari iii. Fallow land iv. Others

1. What is the total area of bamboo plantation?
2. What is the main purpose of bamboo plantation?
3. Food ii. Fodder iii. Medicine iv. Business v. Bamboo artifacts
4. Which part of the bamboo is used for its plantation?
5. Rhizome ii. Culm iii. Branches iv. Seeds
6. How long have you been planted bamboo?
7. What is the total annual income from bamboo resources?
8. Which bamboo artifact is most commonly used in your local area?
9. Doko ii. Dalo iii. Nanglo iv. Furnitures v. Others
10. Is the income from bamboo sufficient for livelihood?
11. If not sufficient, what are other sources of income?
12. Do you know anything about Chure conservation? Yes/No
13. Are you aware about the information that the place you are inhabiting since long time is geographically fragile area?
14. What are the agencies undertaking Chure conservation?
15. Local level ii. National level iii. International level iv. Others
16. What is the participation of people from local level?
17. Low ii. Medium iii. High
18. Do you know about President Chure Tarai Madhesh Conservation and Development Board?
19. What measures do you think is the best way of Chure conservation?
20. Public awareness ii. Plantation iii. Embankment iv. Others
21. What is your opinion regarding the exploitation of Chure resources like gravels, stones, and sand?
22. Positive ii. Negative iii. Others
23. What do you think about Chure conservation in recent context?
24. What are the common problems in your locality?
25. Water resources ii. Forest iii. Farming iv. Land iv. Others
26. How do you take bamboo plantation for Chure conservation?
27. Positive ii. Negative iii. Others
28. What is the perception of the local people regarding bamboo plantation for Chure conservation?
29. Which species of bamboo is said to be more useful and efficient in your locality?

**Questionnaires related to the locally available bamboo species**

1. Which species of bamboo is more common in your locality?
2. Have you taken any training on bamboo plantation?
3. If yes, what kind of training have you participated in?
4. Are the locally prepared or available bamboo artifacts sufficient?
5. How do the local people take the locally available bamboo and bamboo related artifacts?
6. What is the alternate source of bamboo artifacts?
7. What is the quality of bamboo production?
8. Does your bamboo production compete in the local market?
9. If not, what might be the reason?
10. Can more benefit be earned provided the quality of bamboo production is enhanced?

*****The End****
